# Mimicking the Hierarchical Organization of Natural Collagen: Toward the Development of Ideal Scaffolding Material for Tissue Regeneration

**DOI:** 10.3389/fbioe.2021.644595

**Published:** 2021-04-27

**Authors:** Luca Salvatore, Nunzia Gallo, Maria Lucia Natali, Alberta Terzi, Alessandro Sannino, Marta Madaghiele

**Affiliations:** ^1^Department of Engineering for Innovation, University of Salento, Lecce, Italy; ^2^Institute of Crystallography, National Research Council, Bari, Italy

**Keywords:** type I collagen, hierarchical organization, tissue engineering, regenerative medicine, scaffold, collagen processing, fibrous assemblies

## Abstract

Biological materials found in living organisms, many of which are proteins, feature a complex hierarchical organization. Type I collagen, a fibrous structural protein ubiquitous in the mammalian body, provides a striking example of such a hierarchical material, with peculiar architectural features ranging from the amino acid sequence at the nanoscale (primary structure) up to the assembly of fibrils (quaternary structure) and fibers, with lengths of the order of microns. Collagen plays a dominant role in maintaining the biological and structural integrity of various tissues and organs, such as bone, skin, tendons, blood vessels, and cartilage. Thus, “artificial” collagen-based fibrous assemblies, endowed with appropriate structural properties, represent ideal substrates for the development of devices for tissue engineering applications. In recent years, with the ultimate goal of developing three-dimensional scaffolds with optimal bioactivity able to promote both regeneration and functional recovery of a damaged tissue, numerous studies focused on the capability to finely modulate the scaffold architecture at the microscale and the nanoscale in order to closely mimic the hierarchical features of the extracellular matrix and, in particular, the natural patterning of collagen. All of these studies clearly show that the accurate characterization of the collagen structure at the submolecular and supramolecular levels is pivotal to the understanding of the relationships between the nanostructural/microstructural properties of the fabricated scaffold and its macroscopic performance. Several studies also demonstrate that the selected processing, including any crosslinking and/or sterilization treatments, can strongly affect the architecture of collagen at various length scales. The aim of this review is to highlight the most recent findings on the development of collagen-based scaffolds with optimized properties for tissue engineering. The optimization of the scaffolds is particularly related to the modulation of the collagen architecture, which, in turn, impacts on the achieved bioactivity.

## Introduction

Biological tissues are made of cells embedded in a tissue-specific extracellular matrix (ECM). The ECM is an intricate network of macromolecules, including proteins, glycosaminoglycans, proteoglycans, and glycoproteins, which are able to hold a high amount of water (Gelse et al., 2003; Birk and Bruckner, 2011). This provides the ECM with gel-like texture and gel-like mechanical properties. While the ECM was originally thought as a passive system responsible for the structural and functional integrity of tissues and organs, it is now clear that the ECM actually plays a pivotal role as an active regulator of cell phenotype (Birk and Bruckner, 2011; Pawelec et al., 2016). Cell surface receptors recognize specific ligands on the ECM, attach to them, and then transduce signals from the ECM. This type of signaling, including biochemical, topographical, and mechanical cues, regulates various cell functions, such as cellular growth, migration, and differentiation. The interplay between cells and ECM also includes the turnover or remodeling of the ECM by the cells during both normal and pathological conditions through the secretion of several ECM-degrading enzymes, along with the secretion and deposition of ECM components. Very briefly, it can be stated that cells and ECM constitute a highly dynamic and finely regulated system that controls the development, maintenance, and repair of tissues and organs.

Among the ECM proteins, collagen definitely holds the lion’s share. In humans, it is indeed estimated to account for about 30% of the total body protein content (Nimni and Harkness, 1998). The unique fingerprint of collagen molecules is a right-handed triple-helical domain, called tropocollagen, which is formed by three left-handed polypeptide helices, called polyproline-II (PPII) helices (Gelse et al., 2003; Shoulders and Raines, 2009; Birk and Bruckner, 2011). So far, at least 28 proteins having this specific fingerprint have been identified as members of the collagen family, which are differently located within the body and progressively named from type I to type XXVIII according to the time of discovery (Shoulders and Raines, 2009; Birk and Bruckner, 2011). The differences among the collagen types are related to their submolecular, molecular, and supramolecular features, which confer them distinct structural and biological functions (Shoulders and Raines, 2009; Birk and Bruckner, 2011). Type I collagen, which has been the first collagen to be identified (hence being the most widely investigated), is the predominant fibrous component of connective tissues such as the dermis, bones, tendons, ligaments, and cornea, accounting for approximately 70% of the total collagens found in the body (Gelse et al., 2003).

Therefore, it is not surprising that tissue engineering and regenerative medicine, which respectively aim at achieving tissue regeneration *in vitro* and *in vivo*, make extensive use of type I collagen (mostly derived from animal tissues, e.g., bovine) as the biomaterial of choice for the manufacture of cell-instructive scaffolds (Walters and Stegemann, 2014; Terzi et al., 2020). Scaffolds are artificial matrices designed to work as temporary ECM substitutes, i.e., designed to deliver specific structural and biochemical signals to the cells, in order to stimulate the cellular processes involved in tissue regeneration and matrix remodeling. Hence, the scaffold design takes inspiration from the ECM, attempting to mimic as closely as possible the tissue-specific ECM composition and architecture, at various length scales.

Being a major ECM component, type I collagen is intrinsically bioactive and biodegradable. It also possesses low immunogenicity (i.e., the ability to trigger an immune response) and weak antigenicity (i.e., the ability to interact with antibodies) (Bianchini and Parma, 2001; Lynn et al., 2004; Delgado et al., 2017). Furthermore, its versatile processing allows the production of a wide range of tissue engineering scaffolds of various sizes and shapes, e.g., hydrogels, sponges, and fibrous mats (Friess, 1998; Dong and Lv, 2016). However, the cellular interaction with a processed collagen-based scaffold is largely affected by how closely the scaffold is able to recapitulate the structure of native collagen on different length scales (Walters and Stegemann, 2014). In tissues, type I collagen exhibits a peculiar multilevel hierarchical structure, moving from the nanoscale to the macroscale, with each structural level inducing or controlling given biological processes, in addition to providing the tissue (e.g., skin, bone, tendon) with appropriate mechanical strength. Several processing steps, performed during both collagen isolation and scaffold manufacturing, can impact the native collagen assembly to various extents, thus potentially affecting the cellular response and the overall biological activity *in vivo*.

In this review, after presenting the structural hierarchy of type I collagen in tissues and discussing the related biological activity, we address the most recent findings on the development of collagen-based scaffolds with optimized regenerative capability. In particular, our aim is to emphasize that the scaffold optimization goes through a deeper understanding of the structural modifications of collagen induced by processing, along with a detailed analysis of the cellular response(s) elicited by such changes. To this purpose, we first discuss, on a general basis, the effects of the collagen source and the subsequent processing on the scaffold structure and the related biological activity. Then, we focus on the optimization of collagen-based scaffolds for the regeneration of specific tissues (such as skin, cornea, bone, and tendon) by presenting some exemplary attempts to modulate the scaffold biological activity to improve the *in vitro* and/or *in vivo* regenerative performance.

## Hierarchical Structure of Type I Collagen and Related Bioactivity

Together with collagen types II, III, V, XI, XXIV, and XXVII (Table 1), type I collagen is a fibril-forming protein. Fibril-forming collagens are the most widespread in vertebrates, representing about 90% of the total collagens (Gelse et al., 2003).

**TABLE 1:** Fibril-forming collagens found in vertebrates (Shoulders and Raines, 2009; Sorushanova et al., 2019).

**Type**	**Composition**	**Molecular weight α chain (kDa)**	**Distribution**
I	2α_1_(I) α_2_(I)	95	Dermis, bone, tendon, ligament, cornea
II	3α_1_(II)	95	Cartilage, vitreous body
III	3α_1_(III)	95	Skin, blood vessels, intestine
V	3α_1_(V) 2α_1_(V) α_2_(V) α_1_(V) α_2_(V) α_3_(V)	120–145	Bone, dermis, cornea, placenta
XI	α_1_(XI) α_2_(XI) α_3_(XI)	110–145	Cartilage, intervertebral disks
XXIV	3α_1_(XXIV)	–	Bone, cornea
XXVII	3α_1_(XXVII)	–	Cartilage

**FIGURE 1:**
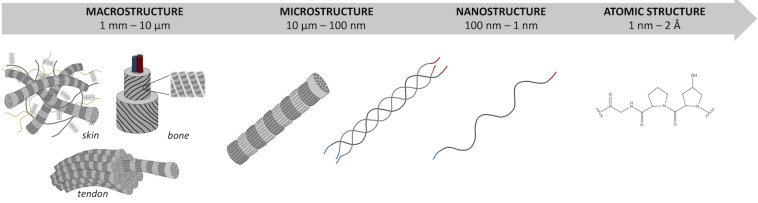
Hierarchical organization of type I collagen skin, tendon, and bone from macroscale to atomic scale.

Briefly, the triple-helical molecular domains (with length ≈300 nm and diameter ≈1.5 nm) assemble to form cylindrical structures known as fibrils (with length ≈μm and diameter ≈100 nm), which are, in turn, organized into fibers (length ≈mm and diameter ≈10 μm). Within tissues (length scale ≈cm), collagen fibers are then either randomly oriented or preferentially arranged in given directions in order to provide the tissue with proper architecture to ensure structural and functional integrity (Figure 1).

Along with this hierarchy at the microscale and macroscale (i.e., at the fibril, fiber, and tissue levels), the single collagen fibrils also show a peculiar hierarchical structure. Similarly to other proteins, four different structural levels can be identified for type I collagen, with one level (i.e., primary) at the atomic/submolecular scale, two levels at the molecular scale (i.e., secondary and tertiary), and one level at the supramolecular scale (i.e., quaternary). These structural levels are concisely described in the following before addressing the tissue architecture. As then summarized in Table 2, some hints on the interplay between the collagen structure and the biological activities are also provided.

**TABLE 2:** Hierarchical structure of fibril-forming collagens: identification of the different structural levels and the related biological activity.

**Structural level**	**Length scale**	**Collagen’s feature**	**Biological activity**
Primary	2 Å–1 nm	Amino acid sequence	• Cell ligand identity
Secondary and tertiary	≈10–100 nm	Molecular conformation • PPII helices–secondary • Triple helix–tertiary	• Cell ligand exposure • Enzymatic resistance and degradation mechanism • Mechanical strength/mechanotransduction* • Antigenicity (for animal-derived collagen used as a biomaterial)
Quaternary	≈1 μm	Packing of molecules • Fibril structure – banding	• Enzymatic resistance • Mechanical strength/mechanotransduction* • Activation of blood clotting
Architecture	≈1–10 mm	Packing of fibrils • Fiber structure • Fiber arrangement or orientation	• Tissue mechanical properties (e.g., stiffness)/mechanotransduction* • Tissue functionality

*By means of mechanotransduction, cells can detect mechanical stimuli from their microenvironment and respond by performing biological processes.

### Primary Structure

The primary structure of collagen refers to the amino acid sequence in the polypeptide chains (called α chains) that make up the collagen molecule and univocally identify the collagen isoform. Type I collagen, for example, is a hetero-trimer consisting of two identical α1 chains and one α2 chain (Table 1), where α1 and α2 chains differ for the amino acid sequence. The primary structure of collagen is tissue-specific, i.e., provides the protein with peculiar biochemical and physical properties that affect cell attachment and other biological functions (Walters and Stegemann, 2014).

In general, collagen can be represented as a repetition of the Gly-X-Y triplet, where glycine (Gly), i.e., the smallest amino acid, occupies every third position in the peptide sequence of the single chain, so as it can be positioned in the core of the triple helix, closely packing the structure. Conversely, side groups in the X and Y positions are exposed to the surface for the sterical interaction with other residues and are frequently occupied by proline (Pro) and hydroxyproline (Hyp), respectively (Shoulders and Raines, 2009). Since each α chain contains about 1,000 amino acid residues, approximately 330 repeating Gly-X-Y triplets per chain can be estimated. In particular, the triplet Gly-Pro-Hyp, unique to collagen molecules, is reported to occur with a frequency of about 12%, while 44% of triplets are in the forms Gly-X-Hyp and Gly-Pro-Y, and the remaining 44% in the general form Gly-X-Y (Sorushanova et al., 2019).

The high content of Gly (higher than 30%) is fundamental to stabilize the triple-helical domain through the formation of inter-chain hydrogen bonds that are perpendicular to the chain axis. These bonds include both direct hydrogen bonds between the backbone -NH group of Gly and the backbone –C = O group of a residue in the X position of the neighboring chain and indirect hydrogen bonds mediated by water bridges, such as those involving the hydroxyl group of Hyp in the Y position (Rich and Crick, 1961; Ramachandran and Chandrasekharan, 1968).

Pro and Hyp contribute to stabilize the triple helix and also stiffen the α chains by preventing rotation around the C-N bond (Sorushanova et al., 2019). It is estimated that about 22% of all residues in the strands of human collagen are either Pro or Hyp (Shoulders and Raines, 2009). Hyp is typical of collagen, thus it is commonly used as a marker to detect and quantify collagen in tissues (Ignat’eva et al., 2007). Remaining residues in the primary structure of collagen include a variety of amino acids that provide the protein tissue-specific bioactivity.

### Secondary and Tertiary Structures

The secondary structure of proteins refers to the peculiar local arrangement of the single polypeptide chains forming the molecule. For collagen, the secondary structure is thus represented by the left-handed PPII helical conformation of the α chains. However, single α chains only have this conformation when associated with the other two chains, with a one-residue staggering, to form a right-handed triple-helical unit (Brodsky and Ramshaw, 1997; Bella, 2016). This triple helix, stabilized by inter-chain hydrogen bonds, represents the tertiary structure of collagen. Since PPII helices would not exist in the absence of the triple helix, the secondary and tertiary structures of collagen are sometimes considered a unique structure (termed “secondary”). It is important to note that the single chains also comprise two short non-helical regions at both the amino (N–) and carboxy (C–) termini, which measure about 9–26 residues (Shoulders and Raines, 2009; Sorushanova et al., 2019) and do not have a repeating Gly-X-Y structure (Kadler et al., 1996). Therefore, the collagen molecule (about 300 nm long and 1.5 nm in diameter) is made of a central tropocollagen unit and two short non-helical ends, called telopeptides.

Zooming into the triple-helical conformation, two models are reported in the literature to describe this structure (Rich and Crick, 1961; Okuyama et al., 2006). The Rich and Crick model (Rich and Crick, 1961), based on fiber diffraction studies on native type I collagen, considers a single inter-chain hydrogen bond per triplet and a 10-fold helical symmetry with a 10/3 pitch (i.e., 10 repeating triplets in three turns, 3.33 triplets/turn), a 28.6-Å axial repeat, and a pitch length of 86 Å. Another more recent model proposed by Okuyama et al. (2006) is based on crystallographic analyses of proline-rich collagen-related peptides (CRPs) and shows a sevenfold helical symmetry with a 7/2 pitch (i.e., seven triplets in two turns, 3.5 triplets/turn), a 20.0-Å axial repeat, and a 60-Å pitch length. In order to match the two models, it has been postulated that the helical pitch of collagen could be 10/3 in proline-poor regions and 7/2 in proline-rich regions. However, it is now acknowledged that a range of helical symmetries can be found in native collagen and among different collagen types (Shoulders and Raines, 2009; Orgel et al., 2014). This variability could also play a role in the interaction of collagen with other biomolecules (Shoulders and Raines, 2009).

Regardless of the helical symmetry, the rope-like triple helix conformation is fundamental to provide collagen with proper biochemical and biophysical properties, including adequate mechanical stiffness, susceptibility to collagenase, and exposure of selected cell ligands, e.g., GFOGER (Gly-Phe-Hyp-Gly-Glu-Arg), to collagen-specific integrin receptors, such as α_1_β_1_, α_2_β_1_, α_3_β_1_, α_10_β_1_, and α_11_β_1_ (Knight et al., 2000; Khew and Tong, 2007; Grover et al., 2012a,b).

The denatured counterpart of collagen, i.e., gelatin, is obtained from the disruption of the triple-helical structure by cleavage of the hydrogen bonds, e.g., by thermal heating (Bigi et al., 2004). The consequent molecular unfolding (i.e., helix-to-coil transition) is mostly irreversible and leads to a loss of structural order, which results in much lower stiffness and much higher susceptibility to proteases compared to native collagen. This is why particular care is needed to avoid denaturation during the processing of collagen-based scaffolds, since tissue regeneration requires the scaffolds to be mechanically stable for a given time length. With regard to cell adhesion, several cell types are able to attach to both native and denatured collagen, suggesting the presence of conformation-independent binding sites that are masked in the triple-helical conformation but exposed to cells upon molecular unfolding. Cellular attachment to gelatin is likely due to the interaction of several integrin receptors, e.g., α_5_β_1_, α_v_β_3_, and α_2_β_1_, with RGD (Arg-Gly-Asp) and DGEA (Asp-Gly-Glu-Ala) ligands (Davis, 1992; Yamamoto and Yamamoto, 1994; Grover et al., 2012a,b).

As for enzymatic degradation, it is worth recalling that the secondary and tertiary structures of collagen are specific substrates for collagenase, thus playing a pivotal role in the ECM (or scaffold) remodeling. Indeed, collagenase is able to cleave the tropocollagen unit through all three α chains, specifically at a single point that is located at about three quarters of the length from the N-terminus (Sunada and Nagai, 1983). The resulting triple-helical fragments then undergo spontaneous denaturation at physiological temperature, thus becoming susceptible to various proteases.

As an additional general note on the biological response to a collagen-based implant, it is also worth mentioning that collagen antigenicity is related to its molecular (secondary and tertiary) structure. Antigenic determinants of collagen can be classified into one of three categories, respectively: (a) helical, i.e., dependent on triple-helical conformation; (b) central, i.e., located within the triple helix, but dependent only on the peptide sequence; (c) terminal, i.e., located in the telopeptides (Lynn et al., 2004). While collagen antigenicity has been attributed mostly to its terminal telopeptides, it is important to note that the location of the major antigenic sites actually depends on the specific donor/recipient species pair being considered, e.g., bovine/human (Lynn et al., 2004).

### Quaternary Structure

In native collagen, rope-like collagen molecules spontaneously assemble to form a three-dimensional crystalline lattice. As mentioned above, collagen fibrils are formed, in which the molecules are quasi-hexagonally packed and super-twisted in a right-handed structure along the longitudinal axis of the fibril (Collins et al., 2019). Interestingly, the super-twisted structure of the fibrils is maintained also through the non-helical telopeptide regions (Shoulders and Raines, 2009).

**FIGURE 2:**
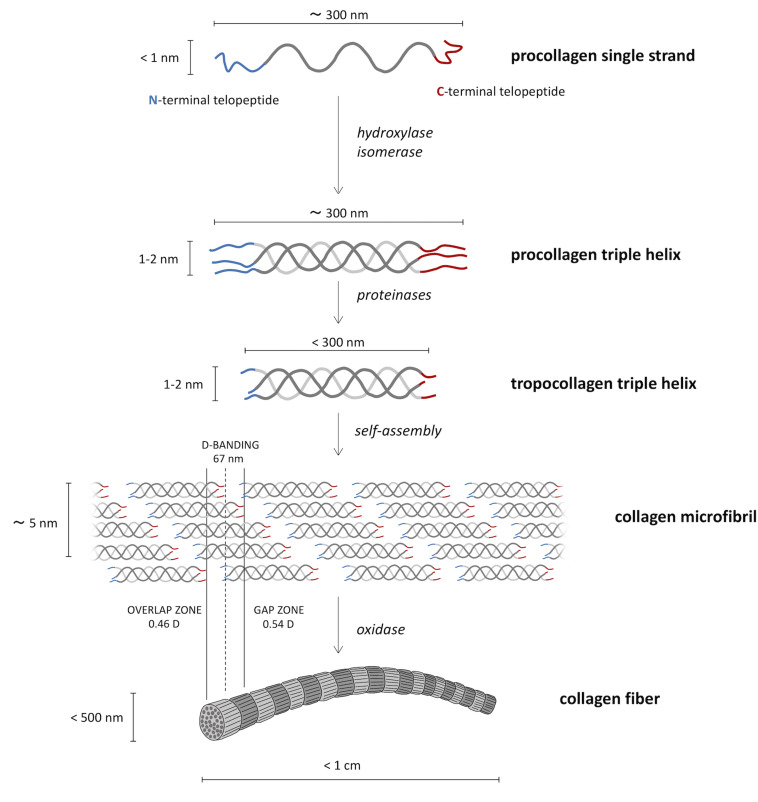
Biosynthetic route from the molecular procollagen to collagen fibers. Three helical left-handed procollagen single strands form a right-handed triple helix of roughly 300 nm in length, named procollagen triple helix. The tropocollagen molecules, resulting from proteinases cut of N- and C-termini, go toward the supramolecular assembly in the so-called collagen microfibrils in the characteristic quarter-staggered form that causes the characteristic appearance of the collagen type I fibrils on the ultrastructural level.

Based on the X-ray diffraction patterns of native collagen, Orgel et al. (2006) proposed a “microfibril” structural model having a triclinic unit cell formed by parts of five different collagen molecules (Collins et al., 2019). This is quite consistent with the simplified microfibril model previously proposed by Hodge and Petruska (1963), who envisaged a bidimensional stack of five collagen molecules aligned parallel to one another with a staggering of about 67 nm (Figure 2). This longitudinal or axial stagger represents the characteristic D-periodicity of the fibrils detected by transmission electron microscopy (TEM), which is the sum of gap and overlap regions between collagen molecules (Figure 2). Indeed, because the molecule length (≈300 nm) is about 4.4–4.5D, a fibril contains a gap or low-electron density regions (about 0.54D long), with some space between the ends of longitudinally lined-up molecules, as well as an overlap or high-electron density regions (about 0.46D long) where side-by-side overlapping of adjacent triple helices occurs (Shoulders and Raines, 2009; Sorushanova et al., 2019). In TEM imaging, gap regions are visualized as the darker ones.

Type I collagen fibrils can be even greater than 500 μm in length and 500 nm in diameter (Shoulders and Raines, 2009; Sorushanova et al., 2019), which suggests the assembly of a huge number of molecules (more than 10^7^). Fibril formation or fibrillogenesis is basically a spontaneous self-assembly process, in which hydrophobic and electrostatic interactions between adjacent molecules occur to minimize the surface area/volume ratio of the final assembly (Kadler et al., 1996; Sorushanova et al., 2019). However, fibril formation can be also cell-regulated, especially in developing or repairing tissues (Kadler et al., 1996). More in general, fibrillogenesis is a tissue-specific process, since different fibril structures and organization are needed to provide the tissue with given functions.

While single triple helices would be unstable at body temperature (i.e., the random coil conformation would be the preferred one) (Shoulders and Raines, 2009), the formation of supramolecular fibrils allows stabilizing the molecular domains. Moreover, this supramolecular assembly is essential to provide collagen with resistance to multidirectional mechanical loading, as well as with enhanced resistance to proteolysis, compared to single molecules.

Although triple helices lacking telopeptides might assemble into fibrils, the C-terminal telopeptides of collagen molecules appear to be involved in the initiation of fibrillogenesis, thus being able to accelerate the process of fibril assembly (Shoulders and Raines, 2009). More importantly, telopeptides play a key role in the mechanical stabilization of fibrils, as they contain sites for both intramolecular and intermolecular crosslinking (Terzi et al., 2020). Subsequently to fibril formation, Lys side chains in the telopeptides are covalently crosslinked forming hydroxylysil pyridinoline and lysil pyridinoline crosslinks between Lys and hydroxylysine residues (Shoulders and Raines, 2009). Covalent crosslinks between triple helices repeat at regular intervals along the fibril, following the fixed stagger pattern. Such a crosslinking provides the fibrils with enhanced strength and stability, as well as higher resistance to proteolysis. Therefore, while telopeptides might not be essential for fibrillogenesis, they are fundamental to strengthen the fibrils *via* the formation of crosslinks (Shoulders and Raines, 2009).

Within the final crystalline lattice, water molecules surround each triple helix, forming a cylinder of hydration that contributes to stabilize the molecular structure (Collins et al., 2019; Terzi et al., 2020). In addition to this “bound” water, free water is also found in the intra-fibrillar spaces. The intra-fibrillar level of hydration clearly affects the intermolecular distance between lateral and longitudinal neighbors (Birk and Bruckner, 2011). Drying of fibrils is indeed known to lead to a shortening of the D-periodicity, as well as a reduction of the lateral packing. For example, the intrafibrillar lateral packing of collagen molecules is reported to vary from 1.6 nm for wet bovine cornea to 1.8 nm for dry rat tail tendon (Terzi et al., 2020). Intermolecular center-to-center distances are also affected by the diameter of the single triple helices, which is in turn dependent on the extent of glycosylation, i.e., the galactosylation and glucosyl-galactosylation of hydroxylysine residues. Differences in the extent of glycosylation are thus a mode of physiological regulation of fibril organization, as it occurs in corneal type I collagen (Birk and Bruckner, 2011).

In addition to determining the structural and mechanical properties of collagen within given tissues, the organization of the collagen molecules into fibrils is actively involved in the process of blood clotting or hemostasis. Indeed, when fibrous collagens, such as type I collagen, are exposed to the blood flow due to injury, platelets recognize specific collagen sequences and interact with the protein, giving rise to the process of blood clot formation (Brass and Bensusan, 1974; Kehrel et al., 1998). Such collagen-induced platelet activation requires both the tertiary and the quaternary structures of collagen (Kehrel et al., 1998).

### Fibers and Tissue Architecture

At the next hierarchical level, collagen fibrils are bound together by interfibrillar proteoglycans that are orthogonal to the fibrils in order to form fibers that have a typical packing distance ≥ 100 nm (Brodsky and Ramshaw, 1997; Shoulders and Raines, 2009). Proteoglycans form crossing bridges between adjacent fibrils by binding collagen at specific sites located at regular intervals of about 60 nm (Terzi et al., 2020). Along with interfibrillar proteoglycans, intrafibrillar proteoglycans are also found, which are oriented along the fibril axis and contribute to the maintenance of the rope-like structure (Terzi et al., 2020). The residual presence of strongly bound proteoglycans, upon the extraction of collagen from animal tissues, is a factor known to affect the antigenicity of the biomaterial (Lynn et al., 2004).

Similarly to the tissue-specific fibril structure, collagen fibers are also tissue-specific and arranged in a peculiar architecture in order to ensure structural and functional ECM performance. For example, in the cornea, all collagen fibrils have small diameters (≈30 nm) and are regularly packed in orthogonal lamellae, which provide not only mechanical stability but also transparency (Birk and Bruckner, 2011). On the contrary, tendon fibrils show a heterogeneous distribution of large diameters (≈50–250 nm), and the fibers are all arranged along the longitudinal axis of the tendon (Birk and Bruckner, 2011; Chen et al., 2017).

In general, heterogeneity is an intrinsic feature of biological tissues, which is present at various length scales and originates at the level of the collagen molecule (Chen et al., 2017). While heterotrimeric triple helices (such as those of type I collagen) are more prevalent than homotrimeric ones (Shoulders and Raines, 2009), it is also important to note that heterotypic fibrils exist, which are assembled from mixtures of two or more fibril-forming collagens (Birk and Bruckner, 2011). Indeed, connective tissues consisting of collagen types I, II, and III (i.e., the quantitatively major fibril-forming collagens) contain minor amounts of collagen types V and XI. The latter are regulatory fibril-forming collagens that are critical for fibrillogenesis and co-assemble with the major fibril-forming collagens to form heterotypic fibrils. The resulting fibril organization and assembly then result in tissue-specific fibril differences (Birk and Bruckner, 2011). For instance, type V collagen is commonly associated with type I collagen in most tissues, especially in the cornea, while type XI collagen is commonly found with type II collagen in cartilage tissues. Additional heterogeneity of the collagen fibrils can also depend on the extent of the lysil oxidase crosslinking (Chen et al., 2017). All of these inherent sources of chemical and structural heterogeneity found at the nanoscale and microscale induce heterogeneity at higher hierarchical levels, thus making the detailed characterization of tissues quite challenging. Heterogeneity at all length scales clearly affects also the mechanical properties of the tissue-specific ECM (Chen et al., 2017).

While the ECM mechanical properties provide structural integrity to tissues, it is worth recalling that they may directly affect the cellular behavior. Upon mechanical loading of the ECM, strain is differently distributed over the distinct hierarchical levels of collagen (from fibers down to molecules) (Gautieri et al., 2011). Cells are continuously exposed to local mechanical cues, arising from their ECM as well as from neighboring cells. By means of mechanotransduction, i.e., a series of molecular processes that transform a physical/mechanical cue into a biological process, cells can perceive a given mechanical stimulus and respond to it (Martino et al., 2018). Mechanical signals, in addition to biochemical and topographical cues, are thus pivotal regulators of cell function. This is the most important reason why cell-instructive scaffolds for tissue engineering should be designed to match as closely as possible the mechanical properties of the target tissue. On the contrary, changes of the compliance of the ECM, which are related to changes of its nanostructure and composition, are commonly associated with the progression of degenerative diseases (Martino et al., 2018).

## Manufacturing of Type I Collagen-Based Scaffolds: Impact on Structure and Biological Activity

Due to the intrinsic complexity and heterogeneity of the ECM at various length scales, the scaffolds currently adopted to recapitulate the ECM composition and structure actually represent oversimplified ECM substitutes. Generally speaking, two strategies can be implemented to produce scaffolds for tissue engineering, respectively a top-down strategy, inherent to the use of decellularized ECMs (Gouveia and Connon, 2016), and a bottom-up strategy, regarding the assembly of scaffolds from molecular building blocks. Whereas the decellularization of the ECMs is successfully applied in some clinical settings [e.g., the use of porcine small intestinal submucosa (SIS) for chronic wounds or the use of decellularized nerve grafts for peripheral nerve injuries], it still poses concerns on reproducibility and sterilization (Garreta et al., 2017). Moreover, although the process of decellularization is claimed to retain the structure of the native ECM, the chemical processes that are involved, if not properly controlled, may have some impact on the collagen structure, in addition to removing key soluble ECM components (Gouveia and Connon, 2016).

Compared to the use of decellularized ECMs, the assembly of scaffolds is much more challenging but advantageous, as it allows building artificial ECM substitutes with tunable and reproducible properties. However, the reconstitution of the native collagen architecture within a scaffold is challenged by the available processing techniques: while the control of the collagen organization on multiple length scales is hard to achieve, the scaffold processing can also impact on the collagen structure on multiple levels. Interestingly, in spite of providing oversimplified ECM substitutes, this close interplay between processing and structure, as detailed in the following, offers the great opportunity to modulate the structure-related biological activity of the scaffolds in order to optimize their capability to induce and sustain tissue regeneration (Table 3).

**TABLE 3:** Impact of processing on the structure-related biological activity of collagen-based scaffolds: overview of the main structural properties (or parameters) modulated by a given processing step and the *in vitro/in vivo* biological properties (or processes) that could be concurrently affected.

**Processing step**	**Modulated structural properties/parameters**	**Modulated biological properties/processes**	**References**
*Choice of the collagen source*	*Thermal stability Enzymatic resistance Mechanical properties*	*Antigenicity/immunogenicity Cell response (via biochemical, mechanical, and topographical cues)*	*Zeugolis et al., 2008b; Parenteau-Bareil et al., 2011; Perez-Puyana et al., 2019; Sorushanova et al., 2021*
Collagen extraction	Triple helix content Telopeptides Fibril network	Antigenicity/immunogenicity Cell response (*via* biochemical, mechanical, and topographical cues)	Delgado et al., 2017; Xu et al., 2017; Bak et al., 2018; Terzi et al., 2020
Aqueous processing of raw material	Banding Triple helix content	Blood clotting Cell response (*via* biochemical and mechanical cues)	Sylvester et al., 1989; Ding et al., 2014; Böhm et al., 2017; Terzi et al., 2018; Stanton et al., 2019
Scaffold shaping/fabrication	Porous/fibrous assembly Macroscopic size and shape Mechanical properties	Cell response (*via* mechanical and topographical cues) Remodeling Vascularization	O’Brien et al., 2005; Antoine et al., 2015; Metavarayuth et al., 2016; Offeddu et al., 2016; Pawelec et al., 2016
Scaffold crosslinking	Thermal stability Enzymatic resistance Mechanical properties	Cell response (*via* biochemical and mechanical cues) Remodeling Foreign body response Vascularization	Chan et al., 2014; Aamodt and Grainger, 2016; Bax et al., 2017; Maternini et al., 2019; Nair et al., 2020a
Scaffold sterilization	Thermal stability Enzymatic resistance Mechanical properties	Cell response (*via* biochemical and mechanical cues) Remodeling	Noah et al., 2003; Wiegand et al., 2009; Proffen et al., 2015; Monaco et al., 2017

*The choice of the collagen source is highlighted (*italics*), since it affects the subsequent processing steps, as well as the final scaffold properties. The generic term “cell response” is used in this table to address the potential effects on multiple cell processes, such as proliferation, migration, and differentiation, with reference to the cell type(s) needed for the regeneration of the target tissue/organ.*

### Collagen Source

The choice of the collagen source represents the first step in the production of collagen-based scaffolds and plays a pivotal role in the subsequent processing, as well as in the determination of the physicochemical, mechanical, and biological properties of the scaffolds (Zeugolis et al., 2008b; Parenteau-Bareil et al., 2011; Perez-Puyana et al., 2019). In general, the collagen used for the manufacturing of scaffolds can be obtained either from recombinant production systems or from animal tissues. Recombinant human collagen can be expressed by both prokaryotic (i.e., bacteria) and eukaryotic hosts (i.e., yeast, mammalian, plant, and insect cells) and offers the potential for consistent collagen production at large scale (Dong and Lv, 2016; Wang et al., 2017). However, recombinant collagen is still far from being similar to native human collagen in a multitude of parameters, since hosts are not able to reproduce the full-length molecule with the native amount of posttranslational modifications (i.e., hydroxylation, glycosylation) (Wang et al., 2017). The incorporation attempts of non-native hydroxylases in *ad hoc* engineered hosts revealed to be unable to adequately hydroxylate collagen molecules (Wang et al., 2017). Although this issue can be overcome by the use of mammalian cells, which require a less extent of genetic manipulation, thanks to the naturally owing of the necessary posttranslational modification apparatus, the yield improvement is very challenging. The genetic manipulation by the introduction of stronger promoter for the increment of collagen production unpairs with the prolyl hydroxylase activity that is thus unable to fully hydroxylate collagen (Wang et al., 2017). Additionally, apart from that, large-scale production is limited by the high cost of mammalian cell culture and the long production time required.

Among eukaryotic systems, it is worth mentioning the outcomes reached by the CollPlant Ltd. company that was able to reproduce a transgenic type I collagen from engineered tobacco plant with hydroxylation levels of both proline and lysine similar to that of human type I collagen (Merle et al., 2002; Stein et al., 2009). Furthermore, they demonstrated how the tobacco-derived type I collagen was able to organize in fibrils, although they did not show the characteristic axial periodicity (Stein et al., 2009). Based on these promising results, CollPlant Ltd. administered two preclinical trials (Shilo et al., 2013; Abir et al., 2020) and one clinical trial^1^ (Identifier: NCT02309307) with plant-derived recombinant human collagen for wound healing and tissue repair.

Despite the potential of recombinant collagens, unresolved issues make them expensive and thus less attractive than animal-derived collagen, which is still the gold standard for use. Therefore, in an attempt to mimic the structure of native collagen as closely as possible, the majority of the collagen currently used in the biomedical field is derived from animal tissues.

Large terrestrial mammals (such as cows, pigs, sheep, and horses) are currently the preferred sources for collagen extraction in the biomedical industry, owing to the high sequence homology with human collagen, as well as the possibility to breed them domestically in large numbers (Silvipriya et al., 2015; Gallo et al., 2020b). In particular, bovine and porcine collagens are the most widely available, being derived from the by-products of the slaughter of beef and pork. Among tissues, tendons and skins are the most commonly used for collagen extraction because of their high collagen content. Up to 85% of the dry mass of the Achilles tendon is reported to be collagen, while in skin, the collagen content is about 70% (Gallo et al., 2020b). The collagen yield from these tissues can be highly variable, depending on the extraction method and on specific characteristics of the animal source (e.g., species, age of animals) but can reach values of 70% or higher (Ghodbane and Dunn, 2016). For research-only applications, collagens from small mammals find also wide use, e.g., the collagen from rat-tail tendon (Davison-Kotler et al., 2019).

In spite of being the current gold standard for biomedical applications, mammalian collagen is not free from risks or limitations. Firstly, it has the potential to induce an undesired immune response; about 2–4% of the population shows allergy to bovine and porcine collagen (Silvipriya et al., 2015). Moreover, it holds the risk of cross-species transmission of infectious diseases, such as foot and mouth disease (FMD) and bovine spongiform encephalopathy (BSE). In this regard, all the sources used for the production of medical-grade collagens are required to be veterinary controlled, traceable, and certified as “disease-free.” Additionally, it is worth noting that the xenogeneic origin often dictates the cultural accommodation of collagen-based products. For example, the use of porcine and equine collagens is not acceptable for Muslim and Jewish people, while the use of bovine collagen is prohibited by the Hindu faith.

In this context, animal sources other than mammals, although showing “immunological distance” from humans (Peng et al., 2010), have been investigated for collagen extraction. While collagen of avian origin (e.g., from chicken skin and feet) may be suitable for biomedical usage (Peng et al., 2010; Li et al., 2017; Perez-Puyana et al., 2019), it still presents the risk of zoonotic diseases, e.g., the avian influenza. On the contrary, collagen from aquatic animals, such as fish, jellyfish, marine sponges, and mollusks, seems safer and more attractive for use (Salvatore et al., 2020a). Indeed, this collagen holds a lower likelihood of transmitting infectious diseases, in addition to being free from any religious or cultural concerns and easily harvested from waste by-products of the fish processing industry. Although the incidence of fish allergy is variable, it is estimated that only 1% of the general population present immune reactions due to fish and fish-based products (Kourani et al., 2019). This further contributes to the common perception that collagen from aquatic animals is safer than collagen from terrestrial mammals. However, the use of this collagen for the production of scaffolds is mainly restricted to research settings, since for most aquatic sources, medical-grade collagen is not yet available. This limitation is due to the need to improve the extraction yields for large-scale production and, most of all, to enhance the batch-to-batch consistency, which is pivotal for clinical implementation (Salvatore et al., 2020a).

In the choice of the optimal collagen source for given tissue engineering applications, the advantages and limitations of each source should be taken into account (Table 4), together with the effects of the source on both the related processing and the final scaffold properties. First of all, the amino acid composition of collagen varies considerably between species (Yamada et al., 2014; Perez-Puyana et al., 2019). The different primary structures directly impact on the biological activity of the protein (i.e., the integrin-binding motifs), as well as the structure at higher length scales and the physicochemical and mechanical properties, including thermal stability, degradation rate, solubility, viscosity, crosslinking, and elastic modulus (Zeugolis et al., 2008b). For example, collagen from aquatic animals demonstrated to have chemical and physical properties significantly different from those of mammalian collagen, such as a lower denaturation temperature (related to a lower amount of imino acids) and a greater degradation rate (Salvatore et al., 2020a). These properties affect the extraction protocols and the subsequent processing phases while requiring the adoption of specific treatments (e.g., crosslinking) to improve the thermal and mechanical stability of the collagen scaffolds for potential clinical use (Salvatore et al., 2020a).

**TABLE 4:** Comparison of the main collagen sources utilized and/or investigated for the production of collagen-based scaffolds.

**Source**	**Immunogenicity/Antigenicity**	**Risk of zoonosis**	**Religious issues**	**Availability/Supply**	Price**	**References**
*Recombinant*	*Low*	*No*	*No*	*Limited*	*Very high*	*Davison-Kotler et al., 2019; He et al., 2021*
*Bovine*	*Low*	*Yes*	*Yes*	*Very high*	*Low*	*Ellingsworth et al., 1986; Charriere et al., 1989; Michaeli and MacPherson, 1990; Lynn et al., 2004; Zhang et al., 2017; Avila Rodriguez et al., 2018*
*Porcine*	*Low*	*Yes*	*Yes*	*Very high*	*Low*	Lynn et al., 2004; Silvipriya et al., 2015; Avila Rodriguez et al., 2018
*Ovine*	*Low*	*Yes*	*No*	Limited*	*Moderate*	*Banerjee et al., 2012; Busra et al., 2019*
*Equine*	*Low*	*Yes*	*Yes*	*High*	*Moderate*	*Adelmann-Grill and Otto, 1987; Bianchini and Parma, 2001; Steel and Twenhafel, 2010; Abdal-Hameed, 2014; Silverstein et al., 2014; Gallo et al., 2020b*
Poultry	Variable	Yes	No	High	High	Pantin-Jackwood et al., 2017; Grønlien et al., 2019
Fish	Variable	Yes (but lower than mammalian and avian sources)	No	Very high	High	Sakaguchi et al., 2000; Kobayashi et al., 2016; Milovanovic and Hayes, 2018; Kourani et al., 2019; Salvatore et al., 2020a
*Jellyfish*	*Low*	*No*	*No*	Limited*	*High*	*Putra et al., 2014; Flaig et al., 2020*

For the sources in *italics*, medical-grade collagen is commercially available, which ensures the safety and the quality of the protein in terms of traceability and batch-to-batch consistency (for scaffold production, fish and poultry collagens have been so far used only in research settings). The limited supply highlighted with (*) indicates a very low number of companies providing that collagen. As for the price (**), a commercially competitive cost of collagen for healthcare uses is in the range 5,000–50,000 USD/kg (Silva et al., 2014).

In addition to the animal species, the specific tissue and the age of the animals are further variables that determine the qualitative features of collagen and collagen-based products (Sorushanova et al., 2021). Indeed, the function-related structural role that collagen covers in tissues, along with the increase of collagen crosslinking with age, deeply influences its hierarchical organization, mechanical properties, and enzymatic resistance (Terzi et al., 2020). With regard to mammalian sources, collagens isolated from skin and tendons exhibit significantly different properties. Tendon collagen fibers are indeed strictly packed and aligned in the main load-bearing direction, in a high hierarchical organization that results in a greater physicochemical, enzymatic, and mechanical stability, while skin collagen fibers, with their anisotropic distribution along Langer lines, are arranged in a loose network that is more easily susceptible to the enzymatic cleavage (Gallo et al., 2020b; Sorushanova et al., 2021). As for the effect of aging, the higher amount of collagen crosslinks with age makes the collagen less flexible and more acid-insoluble (Gautieri et al., 2014).

### Collagen Processing

As already mentioned, the processing of collagen, from the extraction process to the sterilization of the end product, strongly influences its conformational structure and thus its biological, physicochemical, and mechanical performances. In this respect, a recent study by Böhm et al. (2017), who investigated the *in vitro* response of fibroblasts and platelets to collagen matrices obtained from different sources and processing conditions, suggested that the processing of collagen may have a stronger effect on the cell response than the collagen source.

**FIGURE 3:**
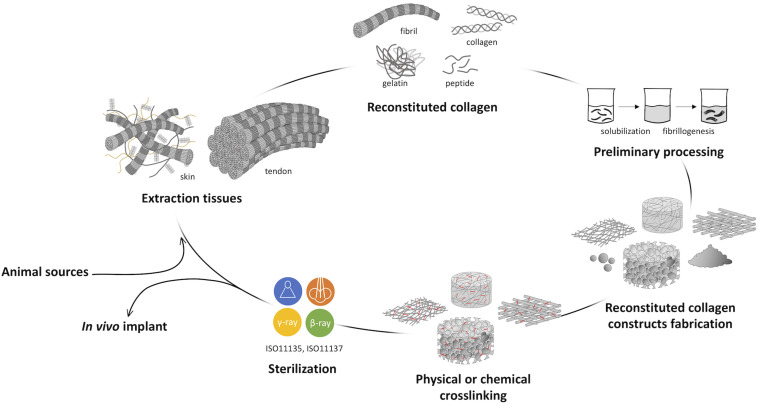
Representative scheme of collagen processing steps from the animal source harvesting to the *in vivo* implant. The development of a collagen-based product can be divided into five macro steps: (i) the extraction or isolation from animal tissues, (ii) the preliminary raw material processing, (iii) the fabrication of the collagen-based product, (iv) its customization by the application of physical and/or chemical crosslinking, and (v) the final sterilization.

As widely known, the development of a collagen-based device could be divided into five macro-steps of manufacturing, including the extraction process, the raw material processing in aqueous solutions, the fabrication of the collagen-based product with given microstructure and macrostructure, its customization by the application of physical and/or chemical crosslinking, and the final sterilization (Figure 3). Terminal sterilization is indeed preferred over aseptic manufacturing due to enhanced repeatability (Monaco et al., 2017). All process parameters of each step could be varied to manufacture a structurally versatile material with varying properties with regard to morphology, mechanics, degradation rate, and behavior in the physiological environment. The possibility to act on all levels allows to finely tune the properties of the collagen-based device in order to achieve the desired final features.

The application of material science and engineering principles to biology for the in-depth understanding of cell interaction with collagen forms led to the identification of several products with specific bioactive properties. Generally, scientific and industrial research focused on the development of three main kinds of reconstituted collagen in the form of native triple helix, gelatin, or peptides of a wide range of molecular weights, according to the application. Although the bioactive properties of gelatin and peptides made them valid raw materials for the cosmetic and food sectors, great relevance has native-like collagen in the biomedical field, attributed to the generally accepted concept that the protein entirely performs its biological function only when it retains its tertiary structure or above. Because of the important role in cell signaling, the collagen triple-helical molecule is characterized by the presence of a high number of integrin binding sites (i.e., the “GFOGER” sequence) that are fundamental for cell adhesion and interaction, proliferation, and differentiation (Davidenko et al., 2016b, 2018). The crucial structural role of collagen in the tissues’ architecture and shape maintenance and its regulatory functions (Gelse et al., 2003; Makareeva and Leikin, 2014) suggested how mimicking the native tissue, by *ex vivo* reorganizing a native-like multiscale and hierarchically organized building blocks, could provide the possibility to finely control the cellular microenvironment and the cell response, enabling functional tissue regeneration. In this attempt, a key role is covered by the investigation techniques employed to correlate collagen structure to its *in vitro* bioactivity. The recent application of new and advanced imaging techniques such as wide-angle X-ray scattering (WAXS), small-angle X-ray scattering (SAXS), TEM, atomic force microscopy (AFM), piezoresponse force microscopy (PFM) allowed to deeply understand the impact of the processing on collagen spatial conformation at the nanoscale level and its interaction with cells.

#### Impact on Submolecular and Molecular Structure

In the manufacturing of a collagen-based product, the extraction of collagen from animal tissues is the preliminary step that, more than all further processing, influences the final properties of the device (Delgado et al., 2017; Xu et al., 2017; Bak et al., 2018). By means of several procedures with specific processing conditions, including exposure times, kind and concentration of chemicals, temperature, and pH, it is possible to obtain different types of reconstituted collagen, from the native protein extracts to peptides, with finely controlled properties at the submolecular and molecular levels. Among them, after the preliminary tissue mincing and milling, a crucial role is covered by the successive chemical and the enzymatic treatments. The exposure time, chemical agent concentration, and temperature deeply control reconstituted collagen structure.

The chemical treatment, usually performed in acetic acid solutions, allows to achieve only a partial solubilization of collagen fibers with a high level of native structure conservation, since intermolecular crosslinked collagen fibrils are not cleaved (Delgado et al., 2017). The enzymatic and non-enzymatic crosslinks present in mature collagen that give it resistance to degradation and chemical cleavage are the main causes of its low solubility in buffers and weak acids usually employed for its extraction. Despite this, long-lasting chemical treatment or high concentrations of chemicals are prevented when the extraction of collagen in native form is required, since long exposure times to acids do not increase collagen yield but damage the protein folding.

For this reason, the enzymatic treatment is almost always routinely performed after the chemical treatment to increase collagen extraction yield (Mocan et al., 2011). The chosen enzyme and its specific cut strictly direct toward the specific type of molecule that will be obtained. Usually, native collagen extraction involves the use of pepsin, an enzyme that cleaves the collagen non-helical telopeptide regions without disrupting its triple-helical structure, thanks to its affinity to unfolded proteins rather than folded. The effective removal of telopeptides can be controlled through the quantification by mass spectroscopy of the tyrosine residues that could be found only in the N-terminal and C-terminal positions. Despite the fact that the enzymatic treatments modify the native length of the collagen by cutting its extremities, the ultrastructural investigation by means of WAXS, SAXS, and Fourier-transform infrared spectroscopy (FT-IR) analyses demonstrated how the finely tuned chemical and enzymatic treatments allowed to retain a partial lateral packing arrangement despite the disruptive treatments of the extraction process (Falini et al., 2004; Terzi et al., 2018, 2019, 2020).

The production of peptides of specific molecular weights and therefore with specific bioactivity occurs through the use of different types of enzymes such as trypsin, chymotrypsin, alcalase, bromelin, collagenase, ficin, Flavourzyme, neutrase, pancreatin, thrombin, papain, and others that have high affinity to collagen in its native form and cut in specific points of the chain (Jung et al., 2014; Hong et al., 2019; Semenycheva et al., 2020). Also in this case, the fine-tuning of digestion parameters (pretreatments, enzyme type and concentration, time, temperature, pH) allows to have peptides with a variable and selective molecular weight distribution and isoelectric points (Zhang et al., 2013; Chi et al., 2015; Hong et al., 2019).

It is essential not to forget the role played by temperature, which must be kept below the collagen denaturation temperature during the whole extraction process when the preparation aims to save the triple-helical structure. On the contrary, the production of gelatin and peptides could involve raising of the working temperature above the denaturation temperature in acidic or alkaline environment with or without enzymes (Gorgieva and Kokol, 2011; Mokrejs et al., 2012).

The length of the collagen fibers could be controlled by tuning the dry matter content during homogenization. “Wet” homogenization (dry matter content in an aqueous system lower than 5%) allows to isolate fibers of several hundred micrometers, while with the “dry” homogenization (10–15% of dry matter contents in an aqueous system), thicker fibers of several centimeters long are achieved (Meyer, 2019).

The influence of the dialysis on collagen and thus on collagen-based scaffold properties and cell behavior was recently investigated by Bak et al. (2018). Supported by Raman spectroscopy and X-ray diffraction analysis, they demonstrated that the presence of acetic acid residues in collagen dialyzed with acetic acid presents a major extent on denatured molecules that directly impacted on mesenchymal stem cell proliferation rate (Bak et al., 2018). Complete elimination of acetic acid residues from collagen by performing dialysis with deionized water is recommended to preserve collagen nativeness and bioactivity.

As previously discussed, the entire extraction process and the physicochemical properties of the final material are strongly dependent on the selected animal source and tissue. More in general, various factors, such as animal age and interspecies variability, make collagen chemical and physical properties highly variable but customizable within a range of values (Gallo et al., 2020b). Keeping in mind all these aspects (i.e., age, tissue, animal, interspecies variability) and the variability of extraction protocols and the sensibility of the instruments and techniques used for collagen characterization, it is not surprising to find literature reports about the mechanical properties (e.g., stiffness) of the different hierarchical levels of collagen, which fall within a very wide range of values, sometimes overlapping (Table 5). Another factor that might lead to discrepancies is the fibril hydration level. Some recent studies confirmed that hydration plays an important role in the elastic modulus of collagen, which in the hydrated state is about 1,000-fold decreased than in the dry state (Yadavalli et al., 2010; Gautieri et al., 2011). However, although high standard deviations are found, it is clear that fiber bundles are the most flexible structural unit (slightly more than whole tissue), four times less stiff than microfibrils and 20 times less stiff than a collagen molecule (Meyer, 2019).

**TABLE 5:** Young modulus of type I collagen in wet state at microscale and nanoscale, calculated as the slope of the tangent to the linear elastic region of stress–strain curves obtained by means of AFM or tensile test.

**Organization level**	**Type I collagen state**		**Young modulus**	**References**
Submolecular and supramolecular	Collagen molecule		3–5 GPa	Lorenzo and Caffarena, 2005; Gautieri et al., 2011
	Collagen microfibril		1–2 MPa	Yadavalli et al., 2010; Gautieri et al., 2011
Microstructure and macrostructure	Film	Not-crosslinked	∼5 MPa	Grover et al., 2012a
		Physically crosslinked	2–5 MPa	Terzi et al., 2018
		Chemically crosslinked	30–300 MPa	Grover et al., 2012a; Nair et al., 2020b
	Electrospun mat	Not crosslinked	–	–
		Physically crosslinked	0.2–1.0 MPa	Drexler and Powell, 2011
		Chemically crosslinked	3 kPa–5 MPa	Drexler and Powell, 2011; Lin et al., 2013; Elamparithi et al., 2016
	Porous membrane	Not crosslinked	∼0.7 MPa	Proffen et al., 2015
		Physically crosslinked	1–5 kPa	Davidenko et al., 2016a; Monaco et al., 2017
		Chemically crosslinked	20–50 kPa	Salvatore et al., 2014; Zhang et al., 2014; Monaco et al., 2017
	Hydrogel	Not crosslinked	0.2–10.0 kPa	Raub et al., 2010; Mason et al., 2013
		Physically crosslinked	0.5–20.0 kPa	Achilli and Mantovani, 2010; Antoine et al., 2015
		Chemically crosslinked	0.4–80.0 kPa	Raub et al., 2010; McBane et al., 2013; Yunoki et al., 2013; Contessi Negrini et al., 2018

*AFM, atomic force microscopy.*

#### Impact on Supramolecular Structure

The conformational organization of the isolated collagen in the form of fiber, fibril, single molecule, or peptide could be further modulated after the extraction process by its preliminary handling prior to its manufacturing by means of further mechanical, chemical, and physical treatments. Several works report that the solubilization step, the solvent used and its concentration, and the homogenization procedures and times strongly affect the collagen supramolecular organization (Gopinath et al., 2014; Bak et al., 2018; Terzi et al., 2018; Zubal et al., 2018; Stanton et al., 2019).

One of the most important aspects in the manufacturing of collagen-based products is to produce devices with reproducible and constant properties. To do this, collagen is usually dispersed in acid aqueous solutions in such a way to form a homogeneous suspension, named “*slurry*.” Obviously, the solvent concentration and type strongly influence its structure and thus its interaction with other molecules. From a biological point of view, the use of acid solutions is reported to disrupt the periodic banding (Figure 2) of the collagen fibrils, thus potentially affecting the related platelet response (Sylvester et al., 1989; Böhm et al., 2017). In addition, despite existing strategies for the removal of excess acid, the presence of non-negligible residues heavily affects the cellular response, as the release of acids is directly responsible for pH changes in the medium, which lead to cell growth slowdown or cell death.

While some types of less packed collagen are soluble in aqueous environment and do not require special precautions, other highly structured ones require a slightly acidic environment as well as the application of mechanical forces to break the strong interaction forces that keep collagen microfibers and do not allow them to uniformly solubilize or disperse in solution. Incomplete solubilization may induce non-homogeneous slurry properties, which would then be responsible for non-homogeneous or non-reproducible properties of the final device. Thus, collagen slurries are usually subjected to several cycles of mechanical homogenization (Terzi et al., 2019; Nair et al., 2020b). The modulation of the homogenization parameters allows to obtain more or less extensive fragmentation of the collagen fibers, thus controlling the supramolecular structure of collagen and, as a result, its bioactivity. In this regard, the tuning of the homogenization process in aqueous solution has been recently found by our team to lead to structural collagen modifications, as well as to control Schwann cell differentiation into myelinating cells (Terzi et al., 2018).

A significant in-depth study was then conducted by Ding et al. (2014), who explored collagen aggregation behavior in acid environment, related to concentration and temperature, to provide information on its use or processing. By means of ultrasensitive differential scanning calorimetry (US-DSC), AFM, and fluorescence techniques, they demonstrated that collagen molecular state was different in solutions with different collagen concentrations. In particular, they found that the denaturation temperature and the corresponding enthalpy slightly decreased with the increase of collagen concentration. The increment of collagen concentration reflects the formation of more hydrophobic interactions among collagen molecules and less water-mediated hydrogen bonds that tend to reduce the stability of collagen and increase the formation of aggregates (Ding et al., 2014). Furthermore, the thermal denaturation of collagen might be influenced by the heat transfer rate: the shorter distance between collagen molecules in aggregates may lead to a higher rate of heat transfer, thus to an easier thermal denaturation (Ding et al., 2014). Additionally, collagen elastic modulus was reported to be strongly influenced by the type and concentration of monovalent salt (e.g., sodium chloride, potassium chloride, ammonium chloride) dissolved into the collagen suspension, which revealed to have a dose-dependent stiffening effect (Grant et al., 2009).

Insoluble collagen, which is usually more structured and more resistant to enzymatic degradation and mechanical load, has the disadvantages to need a more complex manufacturing and to result in a narrower application range. Indeed, the fact that it is soluble in weakly acid solutions, while aggregating and precipitating at physiological values of pH, drastically limits its use for the formulation of hydrogels or injectable suspensions. Some manufacturing “tricks” were developed in order to make native-like ultrastructured insoluble collagen soluble at neutral pH (Li et al., 2015; Zhang M. et al., 2018; Yang et al., 2020a,b). Among these, acylation and desamidation were set up to induce shifts of the collagen isoelectric point to lower values. The acylation of ε-amino group of lysine by means of succinic or glutaric anhydrides makes collagen molecules polyanionic and thus soluble at physiological pH, without affecting its triple-helical structure (Sripriya et al., 2011; Xu et al., 2017). By means of advanced analytical techniques such as XRD, AFM, FT-IR, and circular dichroism (CD), Xu et al. (2017) demonstrated how the collagen conformation was still preserved after acylation. Moreover, this kind of structural modification influences not only collagen solubility but also its resistance against enzymatic degradation, since lysine is one of the recognition residues for the proteases (Sripriya et al., 2011). The biocompatibility of such a treatment has been widely evaluated, and the treatment is currently used for ophthalmic applications due to the high level of thin film transparency (Hadassah et al., 2008, 2010). Less known is the alkaline (i.e., sodium sulfate) treatment of collagen fibrils that progressively hydrolyzes the amide groups of glutamine and asparagine side chains of collagen, resulting in the increase in the number of free carboxyl group and consequently in the sharpening of the isoelectric point (Radhika and Sehgal, 1997; Radhika et al., 1999). While acylation increases collagen thermal stability (Sripriya et al., 2011; Li et al., 2013), desamidation induces a decrement of collagen denaturation temperature (Radhika and Sehgal, 1997). Although the partial destructuration due to the breaking of intermolecular and intramolecular crosslinks between alpha chains and collagen molecules, the alkali treatment apparently does not affect cell behavior, since abnormal cell morphology and cytotoxic phenomena were not detected, confirming the absolute biocompatibility of the so treated collagen (Radhika and Sehgal, 1997; Radhika et al., 1999). However, the lack of in-depth cellular studies on this type of structural modification prevents from defining its long-term effects on cellular behavior.

The last supramolecular structure control, before further processing, could be done by inducing *in vitro* fibrillogenesis. Despite collagen extraction protocols are commonly set up to preserve its native structure as much as possible, the application of mechanical, chemical, and enzymatic treatments brings to a partial destructuration of the strict hierarchical organization of collagen fibrils (Gallo et al., 2020b) that is reflected in fibrils characterized by smaller diameter and length. Thus, the structural organization of the native tissue is not completely preserved in the extracted product. In this regard, some attempts were made in order to *in vitro* reorganize collagen fibrils in fibers that could resemble the natural ones by mimicking the entropy-driven process through which collagen molecules naturally assemble into fibrils.

Fiber packing can be controlled based on solution parameters such as concentration, temperature, pH, and ionic strength (Yadavalli et al., 2010; Harris et al., 2013; Li and Douglas, 2013; Moraes and Cunha, 2013; Ding et al., 2014). The exposure of collagen gels to relatively high temperature (32°C) below the denaturation temperature allows to obtain thicker fibers compared to those at room temperature (Ding et al., 2014). However, at temperatures close to or above the denaturation temperature, the fibers become thinner, suggesting that collagen disaggregation process occurs (Ding et al., 2014). Of key importance is the pH of the collagen suspensions, since it could promote or inhibit the collagen self-assembly process (Yadavalli et al., 2010). However, depending on collagen extraction source and type, different results could be obtained. Additionally, a high number of studies tried to enhance collagen assembly, depending on salt and ion concentration in solution. TEM and AFM revealed that collagen self-assembled into fibrils in the presence of multivalent ions that are able to induce the like-charge attraction and facilitate monomers’ longitudinal registration to form fibrils with the native banding (Li and Douglas, 2013). In particular, Li and Douglas (2013) demonstrated that native fibrils could be reconstituted at pH 7.4 in salts with divalent anions and at pH 9.0 in salts with divalent cations. Some alternative strategies achieved high fibrillation levels by means of the presence of functionalized surfaces. For example, a hyaluronic acid surface coating was found to facilitate the self-reconstitution of collagen, leading to a more mature fibrous network with a twisted structure and enhanced lateral aggregation of fibrils. This fibrous network resulted in enhanced mesenchymal stem cell adhesion and spreading (He et al., 2013).

It is worth noting that, although a partial realignment could be obtained, to date, it is not possible to completely reassemble the extracted collagen fibrils in the ordered hierarchical natural organization.

#### Impact on Microstructure and Macrostructure

As previously mentioned, biological systems are highly hierarchical organized structures constituted by building blocks that enable and regulate the tissue function. To engineer such complex tissues, biomanufacturing tools are necessary to manufacture and model multiscale building blocks. The high versatility of collagen allows it to be manufactured in many different forms whose properties can be adjusted in a broad range. The last processing level that comprises collagen-based device fabrication and sterilization offers another tool for architecture control on the microscale and macroscale. Clearly, the former processing at the submolecular, molecular, and supramolecular level has a strong impact on this step. The manufacturing techniques most frequently used for the production of temporary nanostructured and microstructured substitutes are, but are not limited to, freeze-drying, electrospinning, sol–gel transitions, and the new strategies enclosed in the additive manufacturing. It should not be neglected that the techniques accessible for collagen manufacturing are limited to those that allow handling it at temperatures lower than the denaturation one. However, it is worth noting that, in dry and vacuum conditions, the denaturation temperature can be even exceeded to induce a physical crosslink or to sterilize the collagen-based products.

The porous 3D structure, highly required for tissue engineering applications to promote cell survival, infiltration, and vascularization, could be produced and tuned according to the needs in terms of micro- and macro-architecture (e.g., pore size, shape, orientation, pore wall thickness and roughness) by acting on the manufacturing parameters (Offeddu et al., 2016; Pawelec et al., 2016). Advanced techniques, such as X-ray micro-computed tomography (micro-CT) and scanning electron microscopy (SEM), allowed in-depth investigation on collagen concentration effect on pore features and swelling behavior, revealing an increase of pore wall thickness, closed pore number, and swelling volume with collagen density (Offeddu et al., 2016). Besides to act on the technique, the modulation of the morphological parameters can occur through the addition of solutes to the collagen slurry. By means of SEM imaging investigation, sucrose was found to lead to the formation of smaller pore size, while sodium chloride was found able to stabilize collagen fibrils that became more fibrillar and led to the detection of scaffold pore walls with a more fiber-like surface (Pawelec et al., 2016), in accordance with Grant et al. (2009). Ideally, the porous or fibrous assembly of the scaffold should be tuned to match as much as possible the structural organization of collagen fibers within the target tissue. Indeed, topographical cues are fundamental to promote the desired cell phenotype/differentiation and to direct cell migration, as shown *in vitro* for multiple cell types, e.g., dermal fibroblasts (Poole et al., 2005), mesenchymal (Metavarayuth et al., 2016) and embryonic stem cells (Natale et al., 2020), and corneal keratocytes (Wilson et al., 2012). Moreover, the substrate topography can drive the remodeling of the scaffold, where the secretion of degrading enzymes by the cells and the deposition of new ECM components, in a native tissue-like architecture, may be assisted by the spatial arrangement of the scaffold in a similar pattern (Ahearne et al., 2020).

In this regard, the possibility to develop 3D hydrogel constructs *via* reproducible and flexible molding procedures such as the sol–gel transition is of great interest for the creation of constructs of a variety of shapes and size with a 3D network resembling that of soft tissues. The high number of variables implicated in hydrogel synthesis such as solubilization method, pH and temperature of polymerization, solution components, crosslinker, ionic strength, and collagen concentration allows to control and optimize hydrogel properties in order to better replicate the ones of the target tissue. To this aim, Antoine et al. (2015) developed an empirical predictive model by correlating multiple fabrication parameters in order to facilitate *a priori* design of collagen hydrogels with prescribed properties. An in-depth insight into the features of collagen hydrogels was done by means of a novel optical tweezers-based micro-rheometer technique, which demonstrated the presence of local inhomogeneities in the length scales of 25 μm or larger (Latinovic et al., 2010). The presence of areas of sparse and dense regions reflected the variability of the structural and viscoelastic properties on the microscale, since differences in the elastic properties of the collagen network, of three orders of magnitude higher than the less dense parts, were registered (Latinovic et al., 2010).

Another strategy to resemble an ECM-like architecture is provided by electrospinning, a well-established fabrication technique able to produce submicron non-woven fibers. Various advantages such as high surface–volume ratio, adjustable porosity, and easy surface functionalization made electrospun collagen matrices extremely useful for applications in the fields of tissue engineering, drug delivery, and wound dressing. By means of different strategies, electrospinning allows to manufacture collagen nanofiber networks similar to that present in the ECM in terms of fiber directionality and dimensions. However, native-like collagen suspensions cannot be spun because of inadequate viscosity, solubility, and insufficient solvent evaporation. As such, the electrospinning of gelatin or gelatin/collagen blends is more commonly performed (Monroy et al., 2017; Campiglio et al., 2019).

In the manufacture of implantable devices, key properties are mechanical resistance and enzymatic degradability. Unfortunately, the advantages of collagen biocompatibility, low antigenicity, and high bioactivity collide with disadvantages including low mechanical stiffness and poor resistance to enzymatic degradation. A chance to overcome this issue is offered by the post-fabrication formation of crosslinking bonds between the collagen chains by means of physical, chemical, or enzymatic methods (Table 5). Physical treatments that include ionizing, UV irradiation, dehydrothermal treatment (DHT), and dye-mediated photo-oxidation (Weadock et al., 1996; Gu et al., 2019) allow to achieve low values of crosslink density that restrict their range of application. Hence, a wide range of chemicals such as aldehydes (formaldehyde, glutaraldehyde, acrolein, glyoxal, malondialdehyde, succinaldehyde, dialdehyde starch), isocyanates (hexamethylene diisocyanate), carbodiimides [ethyl-3(3-dimethylamino)propylcarbodiimide], epoxides (1,4-butanediol diglycidyl ether, ethylene glycol diglycidyl ether), and some natural agents extracted from plants (gallic acid, glucose, quinones, genipin, oleuropein) were investigated as crosslinking agents in order to enhance the residence time and the mechanical performances of collagen-based devices (Madaghiele et al., 2009; Salvatore et al., 2014, 2020c; Madaghiele et al., 2016; Snider et al., 2017; Gallo et al., 2018; Terzi et al., 2018; Adamiak and Sionkowska, 2020; Gallo et al., 2020a). Recently, some enzymes such as tyrosinase, laccase, and mostly transglutaminase have also been investigated as non-toxic agents (Gu et al., 2019; Adamiak and Sionkowska, 2020). Depending on the clinical application and the function of implants, crosslinking seems to be advantageous with regard to physical properties such as strength over time, the integration in the surrounding tissue, and the rate and type of remodeling. Each crosslinking method is able to induce different degrees of structural and mechanical stability, which are largely dependent on the crosslinking mechanisms, concentration, and exposure time. Advanced microscopy techniques such as AFM and PFM allowed to investigate the amino acid level modification of collagen piezoelectricity, as well as the ability to modify it using chemical crosslinkers, in order to tailor the electromechanical properties and the related biological response (Nair et al., 2019). Carbodiimide crosslinking of collagen films was found to result in local alignment of collagen fibrils to form thicker fiber bundles, with a perceptible enhanced and localized piezoelectric response; on the contrary, transglutaminase and genipin crosslinked films displayed a non-localized enhanced piezoelectric response (Nair et al., 2019).

Although numerous investigations focus on chemical crosslinking optimization, from a clinical point of view, various authors recommend to avoid chemical crosslinking because of the intensive proinflammatory reaction to crosslinked materials. During resorption, chemical crosslinking affects matrix metalloproteinase (MMP) bioactivity and produce an imbalance in ECM turnover (Aamodt and Grainger, 2016) that results in scar formation. Additionally, resulting collagen degradation fragments are recognized as antigens and amplify the foreign body response (Sandor et al., 2008; Maternini et al., 2019). However, in contrast to the general assumption, Dunn (2012) recently argued that *in vivo* trials often lack comparable conditions and long-term clinical investigations are still missing. One of the most important effects of crosslinkers is their modification of the GFOGER integrin binding sites of collagen helical structure. The hiding or the removal of such recognition sites significantly alters cell differentiation, adhesion, and migration, since cell response is mediated by integrin bonding in a mechanosensing and ligand density-dependent way (Bax et al., 2017). Nair et al. (2020b) demonstrated that the long-term proliferation of human dermal fibroblasts is dependent on the availability of integrin binding sites regardless of the mechanical cues, and that it can be effectively modulated by choosing the appropriate crosslinking. Thus, it is of fundamental importance to consider the mechanism of action and the amount of crosslinker used in order to tune the biological response according to the intended application. In the light of these pieces of evidence, collagen scaffold crosslinking has been customized to direct stem cells toward differentiation (Keogh et al., 2010), organoid formation (Mason et al., 2013; Abbas et al., 2019; Yang et al., 2020c), or to develop 3D cancer models for drug development and testing (Campbell et al., 2017). Interestingly, the crosslinking and stiffening of collagen gels have been also reported to promote the *in vitro* stability of tubular structures, such as blood and lymphatic vessels (Chan et al., 2014).

The considerable advantages offered by crosslinking pushes the research toward the identification of a potential “gold standard” crosslinking protocol for collagen-based devices, to date not yet found. A key problem when comparing different crosslinking treatments is that used protocols often differ greatly. Moreover, additional variations could be ascribed to the different collagens (i.e., extraction source, selected tissue) used as starting materials as well as to their peculiar processing. Indeed, both factors have been shown to have a large effect on crosslinking efficacy and cellular response. While numerous researches demonstrated that mechanical properties and degradation rate could be finely tuned as a function of the crosslinking method, optimized crosslinking methods for collagen-based materials still needs to be developed not only to achieve an adequate balance between stability and functional tissue remodeling but also to affect and control key cell–matrix interactions (e.g., adhesion and migration) required for proper tissue synthesis.

Sterilization of collagen-based devices is the last key process to accurately perform prior to *in vivo* implant. Traditional sterilization methods utilizing heat (autoclaving) are not applicable, since collagen is irreversibly denatured over 60°C (Proffen et al., 2015). Therefore, the most frequently used methods include dry heat (DHS), ethylene oxide (EtO), and high-energy radiations such as beta (β-ray) or gamma irradiation (γ-ray). The major issue in sterilization methods is that all of them induce molecular alteration of the collagen triple-helical structure, with a consequent decrease of mechanical and enzymatic resistance (Noah et al., 2003; Wiegand et al., 2009; Proffen et al., 2015; Monaco et al., 2017). A variety of studies have shown that an unsuitable sterilization process can destroy the structure of collagen, break chemical bonds, and alter physical, chemical, and biological properties substantially (Noah et al., 2003). Therefore, the choice of the sterilization technique is fundamental to preserve the collagen structural and biological role in the ECM-like reconstituted construct.

The treatment with EtO induces changes in the protein structure, a decrement of mechanical properties and a reduction of cell metabolic activity due to the possible presence of toxic residues (Proffen et al., 2015). As well, β-ray can alter the protein structure by directly cleaving the protein chains (Proffen et al., 2015). However, EtO sterilization and β-ray irradiation induce less damage than γ-ray (Parenteau-Bareil et al., 2010; Monaco et al., 2017). The latter indeed is able to break chemical bonds and decrease tensile strength even severely so as to induce a rapid enzyme degradation (Noah et al., 2003). Still, when comparing EtO with β-ray sterilization, the former seems to have less effect on the decrease of mechanical properties (Proffen et al., 2015). The chain scission occurring upon β-ray exposure leads to protein fragmentation and overall increased enzymatic degradability compared to EtO. With respect to EtO and β-ray, DHS treatment resulted in the slowest enzymatic degradation, thanks to the further heat-induced crosslinking that makes the collagen-based scaffold less vulnerable to proteolysis (Monaco et al., 2017).

The modification of the structural properties of the manufactured scaffold could affect the cell response, since the native cell binding sites may be no more available due to collagen triple helix denaturation or chain scissions. With regard to sterilization, conversely to what was expected, Wiegand et al. (2009) demonstrated that γ- and β-ray as well as EtO sterilization did not influence the binding capacity of type I collagen for selected proteases and cytokines associated with non-healing wounds, as no significant loss in the binding affinity for polymorphonuclear elastase, matrix metalloproteinase-2, and interleukin-1β or in the antioxidant capacity was found. However, while these modifications were not reported to significantly affect cell response *in vitro* and *in vivo* (Proffen et al., 2015; Monaco et al., 2017), evidences by Noah et al. (2003) showed a higher proliferation in EtO-sterilized sponges compared to γ-ray-treated ones.

## Optimization of Type I Collagen-Based Scaffolds for the Development of Specific Tissue Substitutes

The manufacturing and the optimization of collagen-based scaffolds for tissue regeneration have been the subject of a large number of studies over the last 40 years. Since the pioneering work by Yannas et al. (1982) on the synthesis of collagen-based sponges for skin regeneration, which dates back to early 1980s, extensive research has been performed *in vitro* and *in vivo*, with the multiple aims of (a) investigating the cellular processes underlying tissue regeneration; (b) understanding the guidance cues (e.g., biochemical, mechanical, and topographical) provided by the scaffold to instruct and orient cells toward tissue regeneration; (c) improving the extent and the quality of induced regeneration in terms of size of the regenerated tissue and restoration of the native tissue structure and functionality. This multidisciplinary research has been enabled and boosted by several technological advancements in both the biology and materials science sectors, such as the optimization of cell culture procedures and the development of more accurate manufacturing technologies and analytical methods.

An exhaustive review on the use of type I collagen-based scaffolds for tissue regeneration would go beyond the scope of this article. Here, we only discuss the optimization of the scaffold structure for improved biological activity by focusing on exemplary cases inherent to the regeneration of skin, cornea, bone, and tendon tissues. Several attempts to develop functional tissue substitutes are described, highlighting, where applicable, critical features due to inappropriate or poor mimesis of the native tissue structure.

### Skin (Dermal Templates)

Skin is the largest organ of the human body, composed of an outer cell-based epithelial layer, i.e., the epidermis, and an inner ECM-based connective tissue, i.e., the dermis. In the dermal ECM, type I collagen is the most abundant fibrous component (80%), along with type III collagen (15%) and elastin (Kaur et al., 2019; Rahmati et al., 2020). Glycosaminoglycans are also found, which form the so-called ground substance, i.e., an amorphous gel in which cells and fibrous components are embedded. The dermis is also highly vascularized and innervated and contains several appendages, i.e., hair follicles, sweat glands, and sebaceous glands, which contribute to skin functionality (Vig et al., 2017; Kaur et al., 2019). Furthermore the dermis is responsible for the different thicknesses of the skin in different parts of the body in relation to the function of the tissue.

Skin constantly deals with intrinsic and extrinsic forces. The effect of mechanical forces is dependent upon the stiffness and biomechanical properties of the skin, which vary between anatomical locations (Wong et al., 2012). As a physical force is applied to the skin, mechanical signals are converted into chemical ones and transmitted to cells in order to trigger a further signaling cascade of responses (Kuehlmann et al., 2020; Urbanczyk et al., 2020). In doing this, ECM components and in particular type I collagen cover an essential role in the transduction of mechanical forces to cell mechanosensors such as cytoskeleton (e.g., actin and transforming protein RhoA), ion channels, catenin complexes, cell adhesion molecules (e.g., focal adhesions and integrins), and several signaling pathways [e.g., Wnt, focal adhesion kinase (FAK)-extracellular signal-regulated kinase (ERK), mitogen-activated protein kinase (MAPK)/ERK] (Alenghat and Ingber, 2002; Kuehlmann et al., 2020; Urbanczyk et al., 2020). Thus, besides the biomechanical aspects, type I collagen is involved in a plethora of additional functions. Signaling by specific receptors that mediate the interaction with collagens, such as integrins, discoidin-domain receptors, glycoprotein VI, or specialized proteoglycan receptors defines adhesion, differentiation, growth, and cell reactivity.

In case of severe injuries, the spontaneous healing response of the dermis consists of a repair process, where the injured site is closed by wound contraction and deposition of an irregular collagenous “scar” tissue. Repair thus leads to the formation of a tissue that is structurally and functionally different from the native one. On the contrary, induced skin regeneration aims to restore the physiologic organ structure and function by using a dermal template (or scaffold) capable of limiting or suppressing spontaneous contraction and scar formation while enabling the synthesis of new dermal tissue (Yannas, 2009; Soller et al., 2012). The understanding of mechanotransduction processes revealed to be important in the manufacturing of a temporary scaffold for tissue regeneration, since mechanical stimuli contribute to alterations in the wound healing process and scar formation (Kuehlmann et al., 2020). Clinical evidence confirmed how the application of mechanomodulatory procedures by the use of a compression device is able to relax skin tension at the site of the wound and effectively reduce scar development, although there remains room for improvement (Lim et al., 2014; Longaker et al., 2014; Orgill and Ogawa, 2014). To date, a multitude of temporary skin substitutes are available for clinical use, which consist of cell-free and/or cell-seeded biomaterial templates (Vig et al., 2017). However, the so-obtained skin is only partially regenerated, as it lacks appendages and does not show the full recovery of biofunctional properties, such as thermoregulation, sensation, and pigmentation (Vig et al., 2017). Therefore, the optimization of dermal templates for improved regeneration is currently the subject of extensive ongoing research.

In the context of skin regeneration, various type I collagen-based templates have been developed and tested in research and/or clinical settings. Groundbreaking studies performed by Yannas et al. (1982) showed that a collagen/glycosaminoglycan-based freeze-dried sponge, crosslinked with glutaraldehyde, could partially regenerate the dermis in animal models and in humans. Although being an oversimplified ECM substitute, this cell-free dermis regeneration template (DRT) (later commercialized as Integra^®^ skin graft) was notably able to induce significant regenerative outcomes. In particular, by modulating some structural parameters of the DRT, it was possible to demonstrate that induced regeneration was strongly correlated to the scaffold ability to delay wound contraction (Yannas et al., 1982). In this regard, two evidence-based mechanisms have been later proposed to explain how the scaffold may hinder contraction, i.e., the reduction of the number of contractile cells (myofibroblasts) in the wound bed and the reduction of the overall contraction forces (Soller et al., 2012). The observed decrease of the number of myofibroblasts in the wound, in the presence of the scaffold (Murphy et al., 1990), could be due to the scaffold ability to non-specifically bind the transforming growth factor beta 1 (TGF-β1), which is a promoter of the myofibroblast phenotype (Yannas, 2009) while preventing platelet aggregation (Sylvester et al., 1989). Indeed, the DRT scaffold is processed by dispersion of bovine tendon collagen in an acid solution, which disrupts the native collagen fiber banding. The consequent inhibition of platelet aggregation may thus lead to inhibition of platelet degranulation and to the concomitant reduction of TGF-β1 from the wound bed (Sylvester et al., 1989). As for the reduction of the contractile force, this may be related to the scaffold ability to bind most of the contractile cells along its randomly oriented collagen struts, thus resulting in ineffective contractile forces, oriented out of the plane of the wound (Yannas, 2005; Soller et al., 2012). Noteworthy, this mechanism would explain why the DRT leads to a maximum delay of wound contraction, i.e., maximum biological activity, only when having specific mean pore size (in the range 20–120 μm, achieved by tuning the freezing rate and temperature in the freeze-drying process) and specific degradation rate (with an *in vivo* residence time of approximately 3 weeks, yielded by tuning the crosslinking) (Yannas, 2005). The requirement of an optimal pore size that suggests an optimal surface area per unit volume may be due to the need for the scaffold to bind an adequate number of contractile cells (O’Brien et al., 2005). Similarly, the requirement of an optimal residence time may be due to the need for the scaffold to persist at least over the period of wound healing (about 3 weeks) during which contraction remains active (Yannas et al., 1989). At the same time, a too long persistence of the scaffold would likely interfere with the synthesis of new tissue. Indeed, the inhibition of contraction is necessary but not sufficient for regeneration, since regeneration also requires tissue formation (Yannas, 2009).

Based on such observations, the following structural determinants of the scaffold biological activity have been identified (Yannas, 2005), which are mostly linked to the scaffold ability to establish direct interactions with the hosted cells: (a) the chemical composition (i.e., the ligand identity); (b) the mean pore size (i.e., the ligand density); (c) the crosslink density (i.e., the ligand duration); and (d) the local spatial arrangement or microstructure (i.e., the ligand orientation). Moreover, collagen banding is an additional structural feature that likely plays a major role in modulating the *in vivo* response. With regard to pore size and crosslink density, it is worth mentioning that the optimal values found by the Yannas team for their DRT are currently used as a gold standard for the development of alternative dermal substitutes. However, the DRT does not present the native collagen banding (it is estimated to have only a 5% residual banding) (Yannas, 2005) while having a microporous sponge-like structure that does not really mimic the fibrillar network of the skin ECM (Dill and Mörgelin, 2020). This relatively poor mimesis of the native collagen architecture may be one of the critical features leading to suboptimal skin regeneration.

Indeed, more recent studies seem to suggest that the preservation of the native tissue structure is highly desirable for improved skin regeneration (Böhm et al., 2017; De Angelis et al., 2018; Tati et al., 2018; Dill and Mörgelin, 2020). When comparing different collagen-based dermal substitutes, all obtained by freeze-drying but varying for the collagen source and the related processing steps (e.g., collagen extraction, aqueous processing, and crosslinking), those with a dermis-like fibrillar network and/or those retaining the native collagen banding have been shown to promote *in vitro* important biological processes involved in tissue regeneration, such as the binding and inactivation of proteases (Tati et al., 2018), an accelerated adhesion, migration and proliferation of cells in the scaffolds (Böhm et al., 2017; Dill and Mörgelin, 2020), an improved cell morphology (Dill and Mörgelin, 2020), and a higher downregulation of the myofibroblast phenotype (Dill and Mörgelin, 2020). Interestingly, in a recent clinical study, an alternative dermal template based on native type I collagen has been shown to lead to accelerated angiogenesis and tissue formation in the short term, as well as to a better clinical outcome in the long term, compared to the Integra^®^ DRT (De Angelis et al., 2018). In that study, the two investigated templates had similar glutaraldehyde-based crosslinking and were both obtained by freeze-drying but mainly differed for the chemical composition (collagen from bovine skin vs. collagen from bovine tendon and glycosaminoglycan) and the related microstructure.

Although requiring further exploration, these findings indicate that the scaffold capability to recapitulate at least some of the structural features of collagen in native dermis may suffice to enhance the scaffold regenerative potential. From a clinical point of view, accelerated angiogenesis is a key factor to improve the outcome of skin regeneration, also considering that the kinetics of angiogenesis is inversely related to the incidence of infections (Yannas, 2009). Therefore, research efforts to improve skin regeneration include the functionalization of cell-free scaffolds with angiogenic molecules, as well as the induction of simultaneous dermis and epidermis regeneration (Oh et al., 2012; do Amaral et al., 2019). For example, it has been recently shown that a DRT added with platelet-rich plasma holds potential for both enhanced vascularization and simultaneous epidermis regeneration (do Amaral et al., 2019). Sophisticated manufacturing techniques, such as 3D bioprinting, are being explored to produce functional full-thickness skin equivalents, starting from cells embedded in collagen-based gels (Ramasamy et al., 2021). While these complex skin substitutes may be particularly useful for *in vitro* studies related to pharmaceutical and cosmetics purposes, they do not appear competitive or easily translatable to the clinical setting, where relatively simple and functional skin substitutes (although yet suboptimal) are already available.

As a final note on skin regeneration, it is worth mentioning that, in spite of the well-known importance of mechanical signals on the cell behavior (e.g., fibroblasts migrating from softer to stiffer substrates) (Kidoaki and Matsuda, 2008), the mechanical properties of the various skin substitutes developed so far have not been widely addressed (Kaur et al., 2019). As an example, the DRT discussed above has a quite low tensile modulus in the wet state (about 500 Pa) (do Amaral et al., 2019), which has not been optimized to match the skin modulus. The measured modulus of human skin is actually quite variable, in the range 0.004–150 MPa, based on body location, age, orientation of the skin sample with respect to the Langer lines, and testing method (Nì Annaidh et al., 2012). The scaffold mechanical properties are thus likely to be critical features that deserve future optimization for an improved regenerative outcome. In this respect, scaffolds with stiffness gradients have been recently proposed to simulate the stiffness gradient between different skin layers (epidermis and dermis), so as to better resemble the mechanical cues that cells in each layer receive from their microenvironment (Rahmati et al., 2020). In addition, the identification of an optimal crosslinking agent is worth a future investigation, considering that toxic glutaraldehyde is still widely used. The effects of various crosslinking agents on both the mechanical properties and the actual availability of cell ligands on the scaffold surface should be properly addressed.

### Cornea

The cornea is a rigid, transparent, and multilayered organ on the eye surface that protects the eye and focuses light onto the crystalline lens, thus ensuring vision. The epithelium is the outermost cell-based tissue, in contact with the external environment, which accounts for ≈10% of the corneal thickness; the stroma (≈90% of the thickness) is the supportive avascular but highly innervated ECM-based tissue mainly responsible for the functional properties of the cornea, such as mechanical strength and transparency; the innermost endothelium is composed of a single layer of endothelial cells that regulate the cornea hydration (Gouveia and Connon, 2016; Ahearne et al., 2020; Mahdavi et al., 2020). Proteoglycans and type I collagen, which are secreted by keratocytes, form the stromal ECM with its unique structural pattern: collagen fibrils, having a diameter of about 35 nm (Raspanti et al., 2018), are regularly arranged in orthogonal layers (or lamellae), in which fibrils lie in a quasi-hexagonal lattice, like a liquid crystal, which enable transmittance of visible light wavelengths and confer suitable mechanical properties to resist the tension exerted by the intraocular pressure (Sibillano et al., 2016). Fratzl and Daxer (1993) also observed that hydration of the fibrils and layers influence corneal mechanical features.

In the anterior part, the fibrils are highly interwoven and dictate the precise curvature of the cornea, which allows to properly focus light for vision (Gouveia and Connon, 2016). Optical clarity of the cornea is also ascribable, in part, to its stromal cells. Indeed, keratocytes, which reside between the lamellae, express corneal crystallins, i.e., proteins that are thought to match the refractive index of the cell cytoplasm with that of the surrounding ECM (Shah et al., 2008).

Considering that damage to corneal stroma results in scarring and impaired or lost vision, great efforts are directed to the development of stromal substitutes *via* scaffold-based tissue engineering approaches, as recently detailed in several reviews (Zhang et al., 2019; Ahearne et al., 2020; Mahdavi et al., 2020). Among the scaffolds proposed for corneal regeneration, those based on type I collagen are largely investigated, in an attempt to mimic the composition of the stromal ECM. Some of the earliest trials to produce corneal substitutes involved the use of collagen-based gels (Germain et al., 1999), sponges (Orwin and Hubel, 2000), and films (Crabb and Hubel, 2008), with the collagen derived from animal tissues, such as bovine or porcine skin or tendon. However, the achievement of suitable strength and transparency, prerequisites for corneal function, soon represented major challenges in the development of practical corneal substitutes. Young’s modulus and tensile strength of the human cornea are reported in the wide ranges 0.1–110.0 MPa and 3–6 MPa, respectively, based on tissue anisotropy, donor variability, and testing methods (Fagerholm et al., 2014; Shih et al., 2017; Ahearne et al., 2020). While several crosslinking methods could be utilized to effectively enhance the stiffness of the collagen substrates without impairing the transparency, the cytotoxicity of the crosslinking agents could limit the cell proliferation (Ahearne et al., 2020). Collagen sponges, obtained by freeze-drying and DHT crosslinking, were found to support the growth of corneal epithelial, stromal, and endothelial cells but also to induce a myofibroblast phenotype of stromal cells over time in culture (Orwin and Hubel, 2000). Interestingly, when comparing DHT-crosslinked collagen sponges and films (with the latter being stiffer than the former), this shift in phenotype was less pronounced on the films, on which cells could grow but not migrate into. This confirmed the utmost importance of both topographical and mechanical cues on cell behavior (Crabb et al., 2006).

Indeed, critical limitations of those earliest corneal substitutes were mostly ascribable to their inability to mimic the dense and sophisticated quasi-hexagonal packing of the collagen fibrils in the corneal ECM, which also led to a mismatch of the mechanical properties. Preliminary studies on the analysis of corneal cell behavior on aligned 2D substrates demonstrated that topographical features could modulate the proliferation of epithelial and stromal cells, in addition to controlling cell alignment, migration, and phenotype (Liliensiek et al., 2006). The development of aligned and dense 3D collagen scaffolds, inspired by the native corneal architecture, has attracted much attention in the last 15 years. With reference to collagen gels, one of the simplest methods to improve the mimesis of native tissue density and mechanical properties is to utilize plastic compression under load. In compressed gels, collagen dehydration and mechanical/physical crosslinking occur, so as to increase both the collagen concentration and the gel stiffness (Cheema et al., 2007; Gouveia and Connon, 2016). Interestingly, cyclic loading has been investigated and found to control the collagen fibril diameter (Cheema et al., 2007). In spite of the simplicity of this approach, compressed gels have been shown to represent a significant improvement over conventional gels for the production of epithelial, stromal, and endothelial constructs (Cheema and Brown, 2013; Levis et al., 2015). Recently, compressed gels containing human corneal stromal stem cells have been reported to suppress scar formation and improve regeneration in a mouse model (Shojaati et al., 2018).

Noteworthy, compressed collagen gels have been utilized to reproduce the native corneal curvature, which is an additional structural requirement deeply affecting cell behavior (Gouveia et al., 2017; Miotto et al., 2019). Indeed, curvature alone has been shown to control the alignment of human corneal stromal cells and their biosynthesis of stromal tissue (Gouveia et al., 2017). While curved substrates are commonly obtained using contact lens molds (Merrett et al., 2008; Gouveia et al., 2017), the self-curvature of compressed collagen gels has been recently induced by patterning the gels with a peptide amphiphile, able to inhibit the contractile activity of stromal cells *via* specific Arg-Gly-Asp (RGD) sequence–integrin interactions (Gouveia et al., 2014). The spatial modulation of the substrate contraction activated by stromal cells has been found to promote the tissue self-curvature over time in culture, then resulting in the formation of a tissue substitute with both native-like curvature and ultrastructure (Miotto et al., 2019).

Other attempts to mimic the native corneal architecture, in order to enhance the biological activity of the tissue substitute, have involved the use of advanced manufacturing techniques that allow controlling the diameter and orientation of the collagen fibers upon deposition. For example, electrospun collagen-based substrates with aligned fibers, in the diameter range 30–500 nm, have been shown *in vitro* to downregulate the myofibroblast phenotype while improving the optical properties of the tissue construct (Phu et al., 2011). However, since electrospinning may have a significant impact on the collagen triple helix conformation, alternative techniques have been explored to produce oriented collagen scaffolds, such as oriented flow casting (Tanaka et al., 2011) and the magnetic alignment of collagen fibrils (Torbet et al., 2007). Electrospun nanofibers based on synthetic polymers have been coupled to collagen gels (Wilson et al., 2012) and compressed collagen gels (Kong et al., 2017), respectively, resulting in downregulation of the myofibroblast phenotype and enhancement of mechanical properties while supporting cell growth.

Bioprinting is an additional manufacturing method that has gained much interest for the production of corneal substitutes due to its ability to control the hierarchical assembly of biological constructs while allowing the fast synthesis of thick and customizable constructs (Isaacson et al., 2018; Sorkio et al., 2018; Zhang et al., 2019; Fuest et al., 2020). Several collagen-based bioinks are being explored to encapsulate and deposit corneal cells upon printing (Isaacson et al., 2018; Sorkio et al., 2018; Duarte Campos et al., 2019; Kim et al., 2019), some of which based on human type I collagen (derived from neonatal fibroblast cells) for better mimesis of the human corneal stroma and higher compatibility with human stem cells (Sorkio et al., 2018). Notably, it has been shown that printed constructs with aligned collagen fibrils and cells can be obtained by properly controlling the shear stress on the bioink upon extrusion (Kim et al., 2019). Compared to non-oriented constructs, the oriented ones were shown to induce the creation of a structure similar to the native cornea upon their remodeling *in vivo* (in a rabbit model) (Kim et al., 2019).

While most substrates investigated for corneal regeneration involve the use of animal-derived collagen, it is worth noting that recombinant human collagens have been explored as safer alternatives for the development of implantable cell-free tissue substitutes. Preliminary studies compared the *in vivo* performance of recombinant type I and type III collagen gels, stabilized *via* carbodiimide crosslinking, in an animal model (Lagali et al., 2008; Merrett et al., 2008). Type III collagen was used due to its ability to form smaller fibrils than type I in an attempt to enhance the fibril packing in the hydrogels. The studies showed that both gel substitutes had similar chemical and mechanical properties, although the ones based on recombinant type III collagen had superior optical clarity. Moreover, both substitutes were effectively integrated into the host corneas 12 months after surgery, maintaining transparency and ensuring regeneration of corneal cells, tear film, and nerve endings (Lagali et al., 2008; Merrett et al., 2008). Following clinical data then demonstrated that the implantation of recombinant type III collagen gels promoted, over 4 years of observation, the endogenous recruitment and regeneration of corneal tissue and nerves, without any rejection episode (Fagerholm et al., 2014). To the best of our knowledge, these findings are the first and only documenting the use of cell-free collagen-based scaffolds for corneal regeneration on humans.

However, despite being so far the most promising devices for a future clinical translation, recombinant collagen gels still need further improvements to optimize their regenerative performance and functional outcome. In particular, some critical features of the gels have emerged in the abovementioned studies, which appear related to their poor mimesis of the native tissue structure and/or properties. First of all, the gels do not have mechanical properties comparable to those of the human cornea (Fagerholm et al., 2014). Although being sufficiently robust for grafting, the gels need particular care for surgical manipulation and suturing, and sutures may result in local surface thinning or indentation of the implants, which compromise the achievable visual acuity (Fagerholm et al., 2014). Moreover, a detailed characterization of the structural and optical properties of the gels showed that these corneal substitutes have a substantially different architecture than the native tissue, as well as a high and undesired transmission of UV light (in addition to visible light), which is potentially damaging to the lens and the retina (Hayes et al., 2015). With regard to the structural properties, compared to the native corneal tissue, the hydrogels were found to show collagen fibers or filaments with smaller diameter and more sparsely distributed, with the absence of stacked lamellae and the absence of fiber interweaving in the anterior part (Hayes et al., 2015). Furthermore, no periodic D-banding was observed, which indicated the lack of native collagen fibrils in the synthetic hydrogels (Hayes et al., 2015). Future structural modifications of the gels, directed to optimize the extent of mimesis of the native tissue (e.g., by implementing more advanced manufacturing techniques for a higher control of the collagen patterning, or by optimizing the crosslinking for suitable strength), may show promise to enhance the therapeutic potential.

### Bone

Bone is a complex, living, constantly changing tissue. With its peculiar architecture and composition, it performs an essential mechanical function besides playing a critical role in diverse metabolic processes mediated by calcium delivery as well as in hematopoiesis. Natural bone is a heterogeneous and anisotropic nanocomposite, the principal components of which are a mineral phase and an organic phase, hierarchically organized into several structural levels from the nanoscale to the macroscale. Thus, bone is principally composed of calcium phosphates (69–80 wt.%, mainly hydroxyapatite and β-tricalcium phosphate), collagens (17–20 wt.%, mainly types I, III, and V), and other minor components (5 wt.%; biglycan, fibronectin, osteonectin, osteopontin, thrombospondin, osteocalcin, alkaline phosphatase, and others) (Filippi et al., 2020; Lin et al., 2020). Type I collagen accounts for the 90% of the total collagen in bone tissue (Lin et al., 2020), and it covers an important role not only in the tissue mechanical supporting but also in biological processes fundamental for bone homeostasis (Lin et al., 2020). Indeed, the lack of type I collagen or mutation of collagen structure (i.e., osteogenesis imperfecta, osteoporosis, osteoarthritis) results in ECM property changes and, more specifically, in the significant reduction of mechanical properties and in the increase of the related fracture risk (Giannini et al., 2014; Lin et al., 2020). Types III and V collagens, present in smaller amounts, regulate the fiber diameter and fibrillogenesis of type I collagen (Garnero, 2015). At the nanoscale level, collagen fibrils assembled from type I collagen molecules (1.5 nm in diameter, 300 nm in length) are infiltered and surrounded by mineral nanocrystals (plate or needle-shaped, 1.5–4.0 nm in thickness) oriented along the axial direction of the fibril (Filippi et al., 2020). Scaling up from the nanostructural to the microstructural frame, collagen fibrils interact with other collagenous and non-collagenous proteins to assemble higher-ordered fibril bundles and fibers organized in 3–7-μm-wide planar sheets, called lamellae (Filippi et al., 2020; Lin et al., 2020). Concentric layers of lamellae of about 200–250 μm of diameter form cylindrical structures that run parallel to the bone long axis, named osteons or Haversian systems (Filippi et al., 2020).

Although bone is constantly remodeled in physiological conditions and presents a strong regenerative capacity, in case of extensive traumas, repairing processes are not able to fully regenerate the injured tissue and surgical interventions are needed. Actually, autograft is the gold standard for repairing defective tissue, but it may lead to donor tissue degeneration and dysfunction (Ma et al., 2021). However, the limited access to autologous bone, donor site morbidity, surgical complications, fitting irregular defect difficulties, as well as inconsistent repair limited its execution (Dewey et al., 2021). On the other side, allotransplantation can cause immune rejection (Ma et al., 2021). Thus, the high number of shortcomings of current treatments headed research for tissue engineering solutions.

Being one of the major bone components and one of the key factors for mineralization beginning and propagation, several collagen-based templates were proposed. Beyond processing, if the regeneration of other tissues is influenced by collagen structure, in the case of bone regeneration, different collagen forms direct osteoblast proliferation and differentiation. In particular, in contrast to soluble and fibrillar forms, denatured forms of type I collagen inhibit the proliferation of osteoblast-like cells and stimulate osteoblastic differentiation, a stage indicated by higher alkaline phosphatase activity and osteopondin expression (Tsai et al., 2010). However, a long-term study showed opposite results, with the delay of proliferation and mineralization mediated by the fibrillar form (Hanagata et al., 2006). The discrepancy between these two studies might be due to collagen sources, processing, and manufacturing but also by the different stages of osteoblastic maturation, a parameter that deeply affects the cell–material interaction.

The arrangement of collagen fibers in bone is much more complex than in other tissues, and it is very difficult to achieve ECM-like bottom-up temporal bone substitutes. To this, oriented collagen fiber scaffolds were not widely investigated for use in bone tissue engineering (Ma et al., 2021). Thus, the challenging goal is to develop scaffolds able to mimic the microenvironment of native bone *in vivo* and to: (i) provide a temporary mechanical support; (ii) have an appropriate porous architecture to promote bone cell migration and differentiation into the scaffold; (iii) encourage osteoinduction; (iv) and enhance osteointegration with the host tissue. Collagen-based freeze-dried scaffolds revealed to have a bone pro-regenerative effect. A huge number of studies investigated the possibility to control the pore size by tuning manufacturing processes in order to develop scaffolds with particular pore features, since osteogenic cells revealed to respond to pore size and orientation (Murphy et al., 1990; Abbasi et al., 2020). While small pore sizes (<100 μm) are associated with the formation of non-mineralized osteoid or fibrous tissue, larger pore sizes (from 100 to 800 μm) are more appropriate to support bone formation (Roosa et al., 2010; Cheng et al., 2016; Liu et al., 2018; Iviglia et al., 2019). Regarding the individuation of the best pore size, several studies reported different ranges but all higher than 100 μm. For Murphy et al. (1990), a pore size of 100–325 μm was optimal. Cheng et al. (2016) reported how a pore size of about 400 μm leads to greater formation of mature bone by promoting vascularization compared to 250 μm. The formation of blood vessels supplies sufficient oxygen and nutrients for osteoblastic activity, leading to the upregulation of osteopontin and collagen type I, and subsequently to a better regeneration level of the bone mass (Cheng et al., 2016).

However, reconstituted collagen physical strength is not high enough to withstand alone the regeneration of large bone defects. Although mechanical and physical properties of collagen can be modified by chemical crosslinking, the combination with stiffer materials allows to significantly improve mechanical properties and bone conductivity. Mineralized collagen scaffolds own better mechanical properties and osteoconductivity and exhibit higher levels of osteogenic gene expression than unmineralized ones (Liu et al., 2011). In this regard, extensive research has been conducted on the fabrication of both intrafibrillar and extrafibrillar mineralized collagen scaffolds from nanoscale to macroscale to promote bone repair, demonstrating solid advantages in intrafibrillar mineralized collagen fibrils (Weisgerber et al., 2015; Weiss-Bilka et al., 2019; Yu et al., 2020). Due to exceptional outcomes, currently, several collagen-mineral scaffolds are approved and commercially available, such as Collagraft^TM^ (Collagen Corp., United States) (Leupold et al., 2006), OssiMend^TM^ Bone Graft Matrix (Collagen Matrix Inc., United States) (Ferreira et al., 2021), and Healos (DePuy Spine, United States) (Carter et al., 2009; Kunakornsawat et al., 2013).

Although good efficacy levels, the lack of an adequate level of osteoinductivity and/or osteoconductivity led to the addition of other components such as metals, in particular iron, manganese (Yu et al., 2020), and strontium (Montalbano et al., 2020; Wei et al., 2021), or cytokines belonging to β-TGF superfamily, namely, bone morphogenetic proteins (BMPs) (Dawson et al., 2009; Lu et al., 2012; Oryan et al., 2014). Among metals, Fe/Mn-enriched collagen-hydroxyapatite scaffolds manifested enhanced adhesion and proliferation of osteoblasts, promoted bone sialoprotein, dentin matrix acidic phosphoprotein 1 expressions, alkaline phosphatase activity, and increased osteogenic-specific gene expression in *in vitro* osteogenic culture and much better *in vivo* bone-regenerating capability compared to scaffolds without Fe/Mn or Healos, a commercial collagen-hydroxyapatite-based scaffold (Yu et al., 2020). As a further example, Infuse^TM^ Bone Graft (Medtronic Sofamor Danek Inc., United States) is a collagen sponge loaded with recombinant human BMP-2 for tibial fracture repair procedures and oral maxillofacial and dental regenerative bone grafting procedures (Dawson et al., 2009). Despite BMP-positive effects, their use is going to be limited due to their high costs, safety, and efficacy concerns, high doses needed, and osteolysis (Oryan et al., 2014).

Recently, 3D printing and the annex potential of computer-aided design and computer-aided manufacturing (CAD-CAM) technology in scaffold according to patient-specific bone defect were investigated (Bahraminasab, 2020). Several biomaterials were printed in blend with collagen such as hydroxyapatite (Keriquel et al., 2017), β tricalcium-phosphate (Bian et al., 2012), polycaprolactone (PCL) (Ciocca et al., 2014), poly(lactide-co-glycolide) (PLGA) (Dewey et al., 2021) in order to improve temporal substitutes’ mechanical strength (Bahraminasab, 2020). Although having promising outcomes, 3D technology is still far from transforming research know-how into clinical products. Printable material properties and the insertion of biomolecules and cells are still challenging in the manufacture of bone tissue scaffold. In the inkjet and extrusion-based bioprinting, the viscosity of the bioink, print speed, nozzle diameter, and dispensing pressure are important factors that affect the mechanical stability of a scaffold and the fate of cells that therefore still need to be standardized (Bahraminasab, 2020).

To date, significant challenges remain in terms of biomaterial strength optimization, osteogenic activity improvement, and ability to fit complex defect geometries. Moreover, mechanical stability and the ability to limit micromotion at the host–implant interface is still a crucial aspect to unravel since osseointegration and bone regeneration directly affect the healing outcome (Dewey et al., 2021).

### Tendon

Tendon belongs to the musculoskeletal system and is principally characterized by highly hierarchically organized type I collagen fascicles (60–85% of tendon dry weight), parallel to the long axis of the tendon (Thorpe and Screen, 2016). Each hierarchical level, from microfibril to fascicles, provides the tissue with non-linear, viscoelastic, anisotropic mechanical properties necessary to afford the strength necessary to translate loads from muscle-to-bone and bone-to-bone (Puetzer et al., 2021). Tendon also contains a range of non-collagenous proteins, present in low amounts, which nevertheless have important functional roles. Proteoglycans (in particular decorin, but also lubricin, versican) represent the 1–5% of tendon dry weight and are the primary component of the matrix interspersing the collagenous units. By binding type I collagen at all organization levels, they cover a particular role in the tissue development and maturation (i.e., fibrillogenesis), and they also contribute to the mechanical properties of the mature tendon (i.e., sliding, lubrication) (Thorpe and Screen, 2016). With regard to glycoproteins, cartilage oligomeric matrix protein (COMP) and tenascin-C (TNC) are known to bind type I collagen, but their function is yet to be established. Lastly, elastic fibers are also found in varying amounts throughout the tendon matrix, with reported concentrations from 1 to 10% of tendon dry weight, with the role to give to the tissue high elasticity, fatigue resistance, and the ability to store and return energy (Thorpe and Screen, 2016). In tendons, fascicles of fibers are longitudinally and transversally oriented, guaranteeing the absorption of the tensile stress, strain limitation, and keeping tendon in the anatomic site during mechanical load. Furthermore, SAXS and WAXS techniques allow to observe additional mechanisms for strain control, i.e., the fibrils sliding and rearrangement that act as buffers against strain (Bianchi et al., 2016). Within tendon, tenocytes are organized in linear arrays aligned with and interspersed between collagen fibers as a 3D network of cells and their processes distributed throughout the tendon (Lavagnino et al., 2015). Cells are in direct contact with collagen bundles: the deformation of tendon ECM from applied loading (i.e., tension, compression, hydrostatic pressure, and fluid shear stress) is transduced to tendon cells. Thus, mechanical signals stimulate cell biochemical pathways and effect cellular processes, such as differentiation, proliferation, tissue development, as in details reported by James et al. (2008) and Lavagnino et al. (2015).

Tendon injuries due to inappropriate physical training, excessive repetitive stretch, or trauma often lead to collagen organization disruption, resulting in loss of function, pain, and decreased mobility (Puetzer et al., 2021). Because of low blood supply, low oxygen consumption, and low metabolism, the self-healing capacity of tendon is very poor (Zhang B. et al., 2018). While small tendon injuries spontaneously heal, larger defects undergo a repair-mediated process generating fibrocartilagenous scar tissue with inferior structural and biomechanical properties that led to the loss of motion and strength along with high rates of injury recurrence (Grier et al., 2017). Current treatments consist of autograft or allograft transplants, but limited availability, risk of immune response, high risk of reinjury, limited long-term function recovery, and donor site morbidity limited their execution (Puetzer et al., 2021; Yuan et al., 2021).

Actually, how to manage damaged tendon is still a great challenge in the clinic. However, tissue engineering offered promising alternatives. Collagen is the principal load-bearing component of tendon ECM and confers high mechanical strength to the tissue. Being theoretically capable of providing the structural properties required for load bearing in musculoskeletal tissues is thus the most employed biomaterial for tendon tissue engineering. As it happens with all other tissues, also in the case of the tendon regeneration, the architecture and the mechanical properties of a collagen-based construct profoundly influence tenocyte response.

The hierarchical organization of collagen fiber is crucial for the mechanical integrity and function of tendons. Therefore, the reproduction of aligned, mechanically strong collagen fibers constitutes an attractive step for the development of bottom-up scaffold able to mimic both the mechanical and biological tendon environment. Several attempts were made by exploiting various principles and techniques with the aim to artificially reproduce the structural organization of type I collagen in tendon. Early attempts were based on the exploitation of the natural phenomenon of the self-assembly, recreating *in vitro* the physiological conditions for the formation of micrometric fibers. To enhance and control this phenomenon, the use of electrochemical control to direct the pH of the collagen fiber assembly microenvironment was used to produce fibrous structures with particularly aligned pattern, microscopically comparable with native tendon tissue (Cheng et al., 2008). Electrostatically and magnetically aligned multilayer collagen membranes characterized by the presence of collagen fibers of 50–400 μm diameter and several inches length were produced by pouring successive layers of collagen gel in electrostatically charged plates and allowing each layer to partially dry before pouring on the next (Gigante et al., 2009). A hybrid attempt, firstly made by Kato et al. (1989) but followed by other researchers, consisted in the reproduction of collagen pseudo-native structure by extruding an insoluble collagen suspension into a neural buffer as a fiber (from 10 to 2,000 μm) and subsequently performing fibrillogenesis *in vitro* (Kato et al., 1989; Zeugolis et al., 2008a). In this regard, it was found that the nature of the collagen type chosen for the extrusion impacted on the final mechanical properties of extruded collagen fiber: compared to insoluble collagen, soluble collagen conferred improved mechanical properties and uniaxial alignment perhaps owing to improved assembly kinetic (Wang et al., 1994; Pins and Silver, 1995). Although these strategies allow to partially recreate the organized structure of tendon collagen, unfortunately, they are not scalable and are therefore limited to *in vitro* applications.

Alternatively, electrospinning was considered an attractive method to fabricate and handle submicron-diameter fibers (100–500 nm) with native D-banding (Matthews et al., 2002), potentially providing the topographical cues that mimic the architecture of native tendon comprising primarily of parallel collagen fibers, which are beneficial for tenogenic differentiation (Cardwell et al., 2014; Yunoki et al., 2015). However, electrospinning of collagen, besides using non-benign solvents (e.g., 1,1,1,3,3,3 hexafluoro-2-propanol, tricloroacetic acid, 2,2,2-trifluoroethanol) that may have a denaturing effect on the triple-helix structure and a deleterious effect on the biological environment, does not allow to reproduce sheets of aligned fibers with native-like mechanical resistance despite the application of different crosslinking methods (Bazrafshan and Stylios, 2019). Thus, the impossibility to replicate the mechanical strength of native collagen and thus to achieve adequate mechanical properties led to the exploration of collagen/synthetic composites. Thus, the enhanced mechanical properties of synthetic materials were coupled with the bioactivity of collagen (Sahoo et al., 2007; Xu et al., 2014; Salvatore et al., 2018; Sensini et al., 2018). A variety of representative synthetic materials, such as poly-L-lactic acid (PLLA), poly(lactide-co-caprolactone) (PLCL), polyurethane (PU), PCL, and PLGA, were successfully electrospun into aligned ultrafine fibers to engineer tendon tissues. The surface modification of aligned ultrafine fibers with bioactive molecules such as collagen allowed to partially overcome the synthetic material poor cellular responses and the lack of cell recognition motif by improving cell attachment, spreading, and proliferation (Sahoo et al., 2007; Xu et al., 2014; Sensini et al., 2018). Moreover, the incorporation of another tendon ECM component such as chondroitin sulfate in collagen-enriched synthetic fibers increased both biomechanical properties and cell response in terms of upregulation of type I collagen, decorin, and fibronectin gene expression, tenocyte alignment and bioactivity, peritendinous adhesion formation, and inflammatory response (Bhavsar et al., 2010; Kinneberg et al., 2010; Caliari and Harley, 2011; Caliari et al., 2012). It is therefore reasonable to assume that the presence of native tendon ECM principal components in electrospun aligned fibers, by recapitulating the biochemical and topographical features of the tendon tissue, could lead to enhanced tenogenic differentiation and *in vivo* tendon regeneration. In this regard, the expression of the tendon-associated gene scleraxis (SCX) and type I collagen gene (COL1) as well as protein tenomodulin was significantly increased (Yuan et al., 2021). Additionally, the expression of TGF-β2, TGFβR-II, and Smad3 indicated the ability to dictate tenogenic differentiation through the activation of the TGF-β signaling pathway (Yuan et al., 2021). Moreover, animal study in rat Achilles tendon repair model corroborated the promoting role of synthetic fiber enriched with type I collagen and chondroitin sulfate in regenerating a tendon-like tissue (Yuan et al., 2021).

All the production techniques for aligned micrometric fibers lead to the formation of such structures with a high degree of organization, very similar to the native one. However, the mechanical properties of these constructs are by no means comparable to those of the natural tissue. For this reason, rather than trying to recreate the nano-fibrous structure, scientific research directed toward the mimicry of the natural mechanical stimuli using collagen scaffolds with a high degree of postprocessing conservation of the biomaterial native structure. Grier et al. (2017) demonstrated that the structural properties (i.e., pore size and collagen fiber crosslinking density) of anisotropic freeze-dried scaffolds with an aligned ellipsoidal pore structure deeply affect tenocyte bioactivity, viability, and gene expression. Anisotropic scaffolds with high crosslinking densities and small pore sizes (about 50 μm) were more able to resist cell-mediated contraction forces, promote tenocyte metabolic activity, and increase the expression of tenogenic genes (i.e., SCX, TNC) (Grier et al., 2017). Also, Sandri et al. (2016) demonstrated the ability of a collagen-based 3D scaffold exhibiting a “core-shell” architecture to induce tendon regeneration in a rat tendon lesion model by the synergistic association of the requirements of strength and bioactivity through the coupling of two components differing in their morphological and mechanical properties: (i) the core component, made of enlaced non-porous collagen-based strips responsible for the mechanical; and (ii) the shell component, made of a highly porous hollow tube exhibiting an aligned and interconnected porous microstructure and suitable cell permeability, able to support and guide tendon cell infiltration and migration during the regenerative process and promote the scaffold integration and vascularization. Moreover, a study by Di Giancamillo et al. (2017) showed how, with the same processing treatment, the collagen extraction source greatly influences the cell response: a porous scaffold made of type I collagen extracted from animal tendon induced a better tenocyte response compared to that one generated by an analogous porous scaffold consisting of collagen extracted from animal skin. The cellular response clearly indicates how collagen extracted from a highly structurally organized tissue such as the tendon, although subjected to disruptive treatments during the extraction process, maintains a greater fiber diameter, length, and alignment than those of extracted from skin, which are thus able to more faithfully mimic the native tendon ECM.

Although the control of scaffold structural and flexural rigidity *via* crosslinking and porosity could provide an ideal framework to resolve structure–function maps by identifying the most compliant levels of anisotropy, stiffness, and nutrient biotransport for tenocyte-mediated scaffold remodeling and long-term phenotype maintenance, the mechanical properties of collagen gels or porous collagen temporal substitutes are vastly inferior to those of tendon. With the aim of improving the mechanical properties of collagen-based scaffolds and above all of providing cells the right mechanostimulation, external forces were applied. To this, physiologic loads required to maintain tendon homeostasis have been identified with both *in vitro* and *in vivo* models since mechanobiology of tendon cells is vital for the maintenance of tissue homeostasis and for the triggering of regenerative processes. Several studies reported how certain loading patterns induce cellular anabolic adaptation of tendon (Hannafin et al., 1995; Cousineau-Pelletier and Langelier, 2010; Magnusson et al., 2010; Galloway et al., 2013). However, overstimulation or hypostimulation led to tendinopathy (Arnoczky et al., 2007). Repetitive loading may lead to overstimulation of tendon cells and to the initiation of a catabolic degenerative response, collagen fibril damage, microdamage, or laxity (Lavagnino et al., 2006, 2014; Arnoczky et al., 2007). Hypostimulation of tendon cells resulting from altered cell–matrix interactions demonstrated to have similar outcomes, such as collagen disruption, hypocellularity, increased matrix metalloproteinase levels, and cell apoptosis (Arnoczky et al., 2007; Egerbacher et al., 2008; Magnusson et al., 2010). A recent work of Zhang B. et al. (2018) reported how a tendon replacement tissue based on a collagen sponge and mesenchymal stem cells by coupled mechano-chemical induction (cyclic stretch and TGF-β1) was effectively able to enhance Achilles tendon regeneration in a rat model.

Efforts to produce aligned hierarchical collagen fibers concentrated on the application of mechanical boundary conditions on collagen hydrogels to guide cells to produce aligned fibrils. In this attempt, a strong influence was covered by collagen concentration: as the concentration of collagen gels increases, cellular contraction of the gel decreases and mechanical properties increase (Puetzer et al., 2015, 2021). Cell-loaded low-density collagen hydrogels (1–5 mg/ml) allowed the production of organized collagen fibrils resembling embryonic tissue (<1 μm in diameter) (Henshaw et al., 2006; Herchenhan et al., 2013). However, the immature form of fibrils and the low density of the material are respectively responsible of inferior mechanical properties and significant contraction, resulting in a lack of therapeutic applications and early rupture (Puetzer et al., 2021). Cell-loaded high-density collagen hydrogel (10–20 mg/ml) instead allowed to develop native sized (30–40 μm diameter) collagen fibers (Puetzer and Bonassar, 2016; McCorry and Bonassar, 2017). Puetzer et al. (2021) was one of the first to reproduce fiber organization similar to bovine juvenile collagen with native fibril banding patterns, hierarchical fiber bundles 50–350 μm in diameter in 6 weeks, and improved tensile properties of about 1 MPa. Although these are some of the largest fibers developed to date in engineered tissues, the mechanical properties were lacking in comparison to native tissue, suggesting a need for further maturation (Puetzer et al., 2021).

Thus, the application of mechanical external forces in addition to the customized structure of the scaffolds allowed adding another piece to the complex development attempt of a multidisciplinary system capable of supporting the regeneration of native tendon tissue. However, the precise magnitude, frequency, and duration of stimulation required for normal tendon homeostasis remain unknown. In addition, despite numerous *in vivo*, *in vitro*, and *ex vivo* studies on tendon mechanical properties and the development of prediction models (Galloway et al., 2013; Fang and Lake, 2016; Herod et al., 2016; Thorpe and Screen, 2016; Thompson et al., 2017; Chen et al., 2018; Carniel and Fancello, 2019; Theodossiou and Schiele, 2019), the precise *in vivo* loading levels required to induce tendon repair are yet unspecified. In this regard, further understanding of the *in vivo* loading of tendons is vital to understand the mechanobiological stimuli required to induce anabolic or reduce catabolic activity (Lavagnino et al., 2015).

## Conclusion

Type I collagen is largely used as biomaterial for the manufacture of cell-instructive devices for tissue engineering and regenerative medicine. Its versatile processing (ranging from the choice of the collagen source and the protein extraction to the fabrication of given devices and several post-fabrication treatments, including crosslinking and sterilization) allows the production of a wide range of scaffolds for tissue regeneration. Notably, the manufacturing affects not only the macroscopic physicochemical and mechanical properties of the final device but also the *in vitro*/*in vivo* cellular response due to changes of the collagen structure occurring at the submolecular, molecular, and supramolecular scales. This strong interplay among processing, structure, and cellular response can be conveniently exploited to optimize the scaffold biological activity and regenerative outcome. In particular, based on the most recent findings on the development of specific tissue substitutes (i.e., skin, cornea, bone, and tendon), the quality of induced regeneration appears to be enhanced when the scaffold exhibits a higher mimesis of the hierarchical organization of native collagen in the target tissue. In this respect, a fundamental role in scaffold optimization is currently played by advanced investigative techniques such as WAXS, SAXS, FT-IR, AFM, PFM, which allow to accurately study collagen structure and its modifications, so as to correlate the direct effects of processing parameters on the microstructure and nanostructure of collagen with macroscopic phenomena, such as the mechanical behavior and the cellular response.

## Author Contributions

All authors listed have made a substantial, direct and intellectual contribution to the work, and approved it for publication.

## Conflict of Interest

The authors declare that the research was conducted in the absence of any commercial or financial relationships that could be construed as a potential conflict of interest.

## Footnotes

1 ClinicalTrials.gov

## Abbreviations

ECM: extracellular matrix
PPII: polyproline-II
Gly: glycine
Pro: proline
Hyp: hydroxyproline
CRPs: proline-rich collagen-related peptides
Phe: phenylalanine
Glu: glutamate
Arg: arginine
Asp: aspartate
Ala: alanine
TEM: transmission electron microscopy
SIS: small intestinal submucosa
FMD: foot and mouth disease
BSE: bovine spongiform encephalopathy
WAXS: wide-angle X-ray scattering
SAXS: small-angle X-ray scattering
AFM: atomic force microscopy
PFM: piezoresponse force microscopy
US-DSC: ultrasensitive differential scanning calorimetry
CD: circular dichroism
micro-CT: X-ray micro-computed tomography
SEM: scanning electron microscopy
DHT: dehydrothermal treatment
DHS: dry heat
EtO: ethylene oxide
β-ray: beta irradiation
γ-ray: gamma irradiation
DRT: dermis regeneration template
TGF-β 1: transforming growth factor beta 1
BMP: bone morphogenetic protein
PCL: polycaprolactone
PLGA: poly(lactide-co-glycolide)
COMP: cartilage oligomeric matrix protein
TNC: tenascin-C
PLLA: poly-L-lactic acid
PLCL: poly(lactide-co-caprolactone)
PU: polyurethane
SCX: tendon-associated gene scleraxis
COL1: type I collagen gene.
